# Killer Meiotic Drive and Dynamic Evolution of the *wtf* Gene Family

**DOI:** 10.1093/molbev/msz052

**Published:** 2019-04-16

**Authors:** Michael T Eickbush, Janet M Young, Sarah E Zanders

**Affiliations:** 1 Stowers Institute for Medical Research, Kansas City, MO; 2 Division of Basic Sciences, Fred Hutchinson Cancer Research Center, Seattle, WA; 3 Department of Molecular and Integrative Physiology, University of Kansas Medical Center, Kansas City, KS

**Keywords:** meiotic drive, gene conversion, genetic conflict, recombination, rapid evolution, meiosis

## Abstract

Natural selection works best when the two alleles in a diploid organism are transmitted to offspring at equal frequencies. Despite this, selfish loci known as meiotic drivers that bias their own transmission into gametes are found throughout eukaryotes. Drive is thought to be a powerful evolutionary force, but empirical evolutionary analyses of drive systems are limited by low numbers of identified meiotic drive genes. Here, we analyze the evolution of the *wtf* gene family of *Schizosaccharomyces pombe* that contains both killer meiotic drive genes and suppressors of drive. We completed assemblies of all *wtf* genes for two *S. pombe* isolates, as well as a subset of *wtf* genes from over 50 isolates. We find that *wtf* copy number can vary greatly between isolates and that amino acid substitutions, expansions and contractions of DNA sequence repeats, and nonallelic gene conversion between family members all contribute to dynamic *wtf* gene evolution. This work demonstrates the power of meiotic drive to foster rapid evolution and identifies a recombination mechanism through which transposons can indirectly mobilize meiotic drivers.

## Introduction

Many genes are maintained in eukaryotic genomes by natural selection because they provide a fitness benefit to the organisms that bear them. Analyses of these genes and their molecular functions constitute the bulk of molecular biology research performed today. However, not all genetic loci provide a fitness benefit to their hosts and some can even be described as parasites. There are many types of parasitic genes, which can comprise large fractions of eukaryotic genomes and can have a substantial impact on shaping genome evolution (Burt and Trivers 2006).

Killer meiotic drive loci are one such class of parasites that can be particularly harmful to fitness. These selfish loci act when heterozygous to destroy the meiotic products that do not inherit them. This killing causes the heterozygote to transmit the meiotic drive locus to up to 100% of the functional meiotic products (Lindholm et al. 2016; Bravo Núñez, Nuckolls, et al. 2018). Killer meiotic drivers have been observed throughout eukaryotes from plants to mammals, even though their selfish behavior generally decreases overall organismal fitness (Lindholm et al. 2016; Shen et al. 2017; Xie et al. 2017; Bravo Núñez, Nuckolls, et al. 2018; Yu et al. 2018). Killer meiotic drivers can directly cause infertility, and biasing allele transmission disrupts the ability of natural selection to choose the best adapted alleles at any linked loci. Genomic loci that suppress drive are therefore predicted to be favored by selection (Crow 1991). Indeed, the activity of many suppressors of meiotic drive has been observed, although only four suppressor genes have been cloned (Tao, Masly, et al. 2007; Grognet et al. 2014; Bravo Núñez, Lange, et al. 2018; Lin et al. 2018).

Detecting meiotic drive and distinguishing it from other phenomena that bias allele transmission can be experimentally challenging, even in the most tractable genetic systems (Burt and Trivers 2006). After establishing the presence of drive loci, identifying the genes responsible often takes years. In addition, the handful of meiotic drive loci that have been cloned in different systems are not homologous to each other, so sequence analysis is generally not useful in identifying novel drivers (Tao, Araripe, et al. 2007; Fishman and Saunders 2008; Phadnis and Orr 2009; Cocquet et al. 2012; Didion et al. 2015; Helleu et al. 2016; Akera et al. 2017; Shen et al. 2017; Xie et al. 2017; Bravo Núñez, Nuckolls, et al. 2018; Dawe et al. 2018; Yu et al. 2018). These factors limit our ability to efficiently analyze the possible presence or impact of meiotic drivers, especially in complex organisms with limited genetic tractability like humans.

Although meiotic drive genes generally do not share DNA sequence homology, they may share certain evolutionary signatures that could guide discovery of novel drive loci from genomic sequence data alone. For example, genetic conflict between drivers and suppressors is predicted to trigger an evolutionary arms race where both sides exhibit rapid evolution (Helleu et al. 2016; McLaughlin and Malik 2017). Similarly, evidence of analogous evolutionary arms races between viruses and host genomes has become widespread and has led to revolutionary insights in virus–host interactions (Daugherty and Malik 2012). However, due to the paucity of cloned meiotic drivers and suppressors, studies of the evolutionary signatures of genes known to cause or suppress meiotic drive are limited (Lindholm et al. 2016).

The *wtf* gene family from *Schizosaccharomyces pombe* offers an exceptional opportunity to study the evolution of meiotic drive systems (Lopez Hernandez and Zanders 2018). The genomes of *S. pombe* isolates contain more than 20 *wtf* genes, some of which are known to be killer meiotic drivers (Hu et al. 2017; Nuckolls et al. 2017). The characterized drive genes are predicted to encode transmembrane proteins, but there are no obvious orthologs outside of *S. pombe* and the complete molecular mechanisms of drive are unknown. However, the characterized driving *wtf* genes use alternate transcripts to generate both an antidote and a poison during gametogenesis. The poison acts on all gametes, whereas the antidote remains within *wtf*+ gametes. The combined action of the poison and antidote proteins results in the preferential death of the *wtf*− gametes generated by *wtf*+/*wtf*− heterozygotes and therefore preferential transmission of *wtf+* alleles (Hu et al. 2017; Nuckolls et al. 2017).

The driving *wtf* genes impose significant fertility costs on their hosts and severely limit the ability of *S. pombe* isolates to reproduce sexually (Zanders et al. 2014; Hu et al. 2017). Novel genes or genetic variants that can suppress the action of *wtf* drivers are expected to promote fitness and should be favored by natural selection (Crow 1991). Indeed, we recently identified a suppressor of a killer *wtf* drive gene. Interestingly, this suppressor, *wtf18-2*, is a member of the *wtf* family and likely evolved from a *wtf* driver (Bravo Núñez, Lange, et al. 2018).

In this work, we assemble and annotate the *wtf* genes from two *S. pombe* isolates and compare them with the *wtf* genes of two previously published *S. pombe* isolates (Wood et al. 2002; Hu et al. 2017). We classify the *wtf* genes into possible functional groups based on previously characterized genes. In addition, we greatly extend previous evolutionary analyses of the *wtf* gene family (Bowen et al. 2003; Hu et al. 2017). Consistent with their engagement in molecular arms races, we show that *wtf* genes exhibit rapid evolution. In fact, *wtf* genes are among the most rapidly evolving genes in *S. pombe*. We show that intact *wtf* gene numbers vary between isolates. Moreover, we show that syntenic *wtf* genes often have much lower sequence identity than is typically observed between isolates. We show that homologous recombination, repeat expansion and contraction, and amino acid substitutions all contribute to the diversification of the *wtf* gene family. This work provides a case study for the evolutionary dynamics between selfish genes and their suppressors and supports the idea that signatures of rapid evolution could guide the discovery of novel drive loci.

## Results

### Correcting *wtf* Gene Annotations in the *Sp* Reference Genome

The PomBase database provides annotated gene structures for 25 *wtf* genes, of which five are annotated as pseudogenes (Wood et al. 2012; McDowall et al. 2015). However, our previous analyses of the *Sp wtf4*, *Sp wtf13*, and *Sp wtf18* loci revealed that the annotated splice sites were inconsistent with published long-read RNA sequence data (Nuckolls et al. 2017; Bravo Núñez, Lange, et al. 2018). We therefore reevaluated the remaining *Sp wtf* gene annotations using long-read RNA sequence data (supplementary fig. 1, Supplementary Material online) (Kuang et al. 2016). We found that our predictions were consistent with the PomBase annotations for 14 *wtf* genes but different for the remaining 11 genes. Our results matched those of Hu et al. (2017) who predicted the coding sequences computationally. In the updated annotations, four *wtf* genes that were previously predicted to be intact (*Sp wtf6*, *wtf8*, *wtf12*, and *wtf17*) are truncated by early stop codons (based on homology to other *wtf* genes). These genes join *wtf1*, *wtf2*, *wtf3*, *wtf22*, and *wtf24* as likely pseudogenes.

### *wtf* Gene Numbers Vary Greatly between *S. pombe* Isolates

The molecular arms race model predicts that genes in conflict, such as meiotic drivers and their suppressors, will evolve rapidly in order to outcompete one another (McLaughlin and Malik 2017). Gene duplication is a commonly used evolutionary strategy to facilitate rapid diversification and has been observed in the context of virus–host arms races (Daugherty and Malik 2012). The large number of *wtf* loci in the reference *S. pombe* genome assembly (25 genes, including pseudogenes) is consistent with a similar scenario occurring within the *wtf* family. In addition, previous limited analyses revealed differing numbers of *wtf* genes between different *S. pombe* group isolates (Hu et al. 2017; Nuckolls et al. 2017). To more globally test the possibility that recent *wtf* gene duplications or deletions have occurred in the *S. pombe* group, we first determined whether *wtf* gene numbers are dynamic between isolates.

The reference S*. pombe* isolate (972, isolated in France in 1921) was sequenced using extensive physical mapping and Sanger chemistry to yield an excellent assembly including the complete sequences of the *wtf* genes (Wood et al. 2002). Complete *wtf* sequences are also available for the CBS5557 isolate (collected in Spain, reported 1964) which was sequenced using long-read PacBio technology (Hu et al. 2017). The genomes of over 150 additional *S. pombe* isolates have also been sequenced, but those studies used paired-end 100-bp Illumina reads with standard insert sizes (∼300 bp) (Hu et al. 2015; Jeffares et al. 2015; Jeffares 2018). Due to the repetitive nature of the *wtf* genes and the fact that they are often flanked by repetitive Tf transposons or Tf long terminal repeats (LTRs), the sequences of most *wtf* loci could not be reliably determined in those genomes where only short reads were available.

**Fig. 1.**
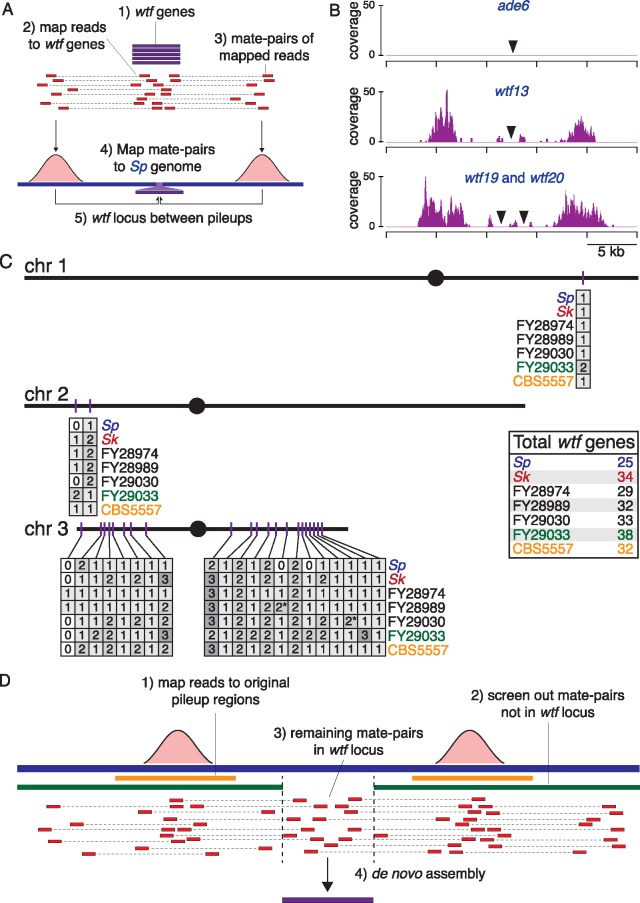
A genomics approach identifies and assembles *wtf* gene sequences. (*A*) Schematic of the strategy we used to identify *wtf* gene locations. (*B*) Examples of three *Sp* loci are shown to illustrate how read pileups (from strategy described in [*A*]) flank loci with zero, one or two *wtf* genes. In each plot, the *x* axis shows relative position in the *Sp* reference genome, and the *y* axis shows the number of reads mapping to each base. (*C*) A map of *wtf* gene distribution in seven isolates of *Schizosaccharomyces pombe*. The map shows the three chromosomes of the *Sp* karyotype, although this karyotype is not shared by all isolates. The inset box indicates total *wtf* gene numbers (including pseudogenes) in each isolate. The numbers for FY28974, FY28989, and FY29030 (in black) are estimates because we did not assemble all *wtf* loci in those isolates. The * denotes the *wtf* genes found in a duplicated region. (*D*) Schematic of the strategy we used to assemble *wtf* gene sequences in *Sk* and FY29033.

To overcome this challenge, we sequenced five *S. pombe* isolates and the reference isolate using “mate-pair” libraries to capture pairs of 150-bp reads separated by 5–8 kb in the genome (supplementary fig. 2, Supplementary Material online). With this large insert sequencing approach, the distance between mate-pair reads is large enough that when one read of the pair falls within a repetitive *wtf*, the mate often falls in unique genomic sequence (fig. 1*A*). This allowed us to match *wtf* reads with their cognate genomic locus, even for *wtf* genes that share very high sequence identity. We sequenced to >80× coverage a derivative of the *S. pombe* reference isolate (which we will abbreviate as *Sp*), FY28974 (collected in Brazil in 1996), FY28989 (collected in Africa in 1921), FY29030 (collected in Indonesia in 1949), FY29033 (collected in Indonesia in 1923), and *Schizosaccharomyces kambucha* (abbreviated *Sk*, isolated in the United States, reported in 2002) (Singh and Klar 2002; Wood et al. 2002; Jeffares et al. 2015). *Sk* was historically given a different species name because it is reproductively isolated from *Sp*, but it is no more diverged from *Sp* than other isolates of the *S. pombe* group (Singh and Klar 2002; Rhind et al. 2011; Zanders et al. 2014). Like all isolates classified as *S. pombe*, all isolates analyzed in this work are all very closely related: They are estimated to have diverged from each other within the last ∼2,300 years and share on average >99% DNA sequence identity (Jeffares 2018).

To identify genomic loci that harbor *wtf* genes in each isolate, we first used our sequence data to select all read pairs in which one of the reads aligned to one or more of the 25 *wtf* genes in the reference genome (abbreviated as *Sp* here). We then isolated mates of those *wtf* reads, aligned them to the *Sp* reference genome, and visually analyzed regions where multiple *wtf* mate reads mapped (“pileups”). This yielded a map in which each *wtf* locus is flanked by pileups of mate reads that map uniquely in the genome (fig. 1*A* and *B*). To verify this approach, we applied it to the *Sp* data and accurately detected all *wtf* locations. We further observed that *Sp* loci containing a single *wtf* gene were typically flanked by ∼2.2-kb-wide pileups, slightly wider than the typical genomic width of a *wtf* gene (average 1.2 kb). *Sp* loci encoding two *wtf* genes were flanked by wider (∼4.4 kb) pileups (fig. 1*B* and supplementary fig. 3, Supplementary Material online). These data suggested that we could use the presence and width of such pileups to identify *wtf* loci and copy number genome-wide.

We then used this approach to identify *wtf* loci in each of the five isolates we sequenced and to estimate how many *wtf* genes each locus contains (fig. 1*C*). In *Sk* and FY29033, these estimates were confirmed (and in a few cases corrected) by assembly of the *wtf* loci from the mate-pair reads and by Sanger sequencing of a group of genes (described below). Unlike in *Sp*, in each of the other isolates we found a few loci flanked by even wider pileups (up to∼7.5 kb), suggesting that these loci each contain three *wtf* genes (supplementary fig. 3, Supplementary Material online). This inference was confirmed by assembling four such loci.

At most of the loci we detected, we observed a symmetrical pair of pileups that were ∼2.2 or ∼4.4 kb wide that clearly suggested one or two *wtf* genes within the locus. Some loci, including most of those with three *wtf* genes, showed more complicated or misleading patterns (examples are shown in supplementary fig. 4, Supplementary Material online). At asymmetric pileups, we used the widest pileup to predict the number of *wtf* genes. Assembly of these regions with complicated pileup patterns in *Sk* and FY29033 revealed that these inconsistencies were due to transposon insertions near the *wtf* loci that were not present in the reference genome. For example, a transposon insertion led to the two genes at the *wtf2* locus in *Sk* showing a pileup pattern typical of a one gene locus, and an additional transposon led to the three genes at the *wtf10* locus in *Sk* to show a pileup pattern typical of a two gene locus (supplementary fig. 4, Supplementary Material online). The underestimates in our FY29033 and *Sk* gene number predictions (at 3 out of 46 loci) were detected during the assembly of those loci to obtain *wtf* gene sequences (below). As we did not assemble all *wtf* loci in FY28974, FY28989, and FY29030, there could be similar uncorrected underestimates of *wtf* gene numbers in those isolates. In addition, our method would be unable to detect more than three tandem *wtf* genes because the locus size exceeds the insert size between our mate-pair reads (supplementary fig. 2, Supplementary Material online). Although we did not observe loci with more than three tandem *wtf* genes in genomes with assembled *wtf* loci, this limitation could also lead to an underestimate of *wtf* gene numbers in the genomes where we did not perform de novo assemblies.

Our mate-pair pileup approach could also miss additional *wtf* gene copies if they were found within larger recently duplicated regions of the genome. To look for such *wtf* genes, we aligned all sequence reads for each isolate to the *Sp* reference genome and looked for regions containing *wtf* loci where sequencing coverage was at least twice as high as the rest of the genome. We found two duplicated regions that include a *wtf* gene (supplementary fig. 5, Supplementary Material online). In FY29030, there is a ∼14-kb duplication of the *wtf23* region (between chromosome 3 reference genome positions 2,145,417 and 2,159,329). In FY28989, there is a ∼95-kb duplication of the *wtf33* region between positions 1,838,980 and 1,933,773 on chromosome 3 (supplementary fig. 5, Supplementary Material online). These duplications both appear to be very young, as we do not detect increased sequence variation in those regions compared with the flanking sequence. We therefore conclude that FY29030 contains two nearly identical copies of *wtf23* and FY28989 contains two nearly identical copies of *wtf33* (indicated with asterisks in fig. 1*C*).

After identifying all the genomic loci encoding *wtf* genes in the five isolates, we combined our data with the previously identified *wtf* landscapes in CBS5557 and *Sp* (McDowall et al. 2015; Hu et al. 2017). Altogether, we found that the total number of *wtf* genes (including pseudogenes) varied greatly between isolates, ranging from 25 in *Sp* to 38 in FY29033 (fig. 1*C*). Each locus can contain between zero and three *wtf* genes. Overall, the locations of *wtf* genes were quite similar between isolates: We found only four *wtf* loci that were not shared among all isolates. Most of the variation in *wtf* number between isolates can be explained by expansion/contraction of *wtf* gene numbers within each locus (fig. 1*C*), although without a clear outgroup it is unclear what the relative contributions of duplications and deletions are. Given that all isolates encode at least one *wtf* gene at 20 shared loci, it is likely that the ancestral genome of these isolates contained at least 20 *wtf* genes.

### Assembling *wtf* Genes from *Sk* and *FY29033* Yields Many Unique Gene Sequences

Within *Sp*, there is extensive sequence diversity among the *wtf* genes. Some, like *Sp wtf4* and *Sp wtf13*, are very similar (>90% amino acid identity), whereas others, like *Sp wtf4* and *Sp wtf7*, are not (<30% amino acid identity). We wanted to know whether the gene repertoire of *Sp* reflects the full range of *wtf* diversity, or whether it instead represents a limited sample. To test this, we used our sequencing data to assemble all *wtf* genes from two additional genomes, *Sk* and FY29033. We assembled each *wtf* locus separately, first selecting all read pairs in which one of the reads aligned to a unique *wtf*-flanking region (i.e., the pileup regions discussed above; fig. 1*A*, *B*, and *D*). We then assembled those read pairs to generate a contig with the *wtf* gene(s) in the center (fig. 1*D*).

To validate this approach, we also used it to assemble *Sk wtf* genes we had previously Sanger sequenced (*wtf4*, *wtf5*, *wtf6*, *wtf13*, *wtf18*, *wtf21*, *wtf26*, and *wtf28*; Nuckolls et al. 2017; Bravo Núñez, Lange, et al. 2018). We also Sanger sequenced *Sk wtf9*, *wtf17*, *wtf19*, *wtf20*, *wtf23*, *wtf27*, *wtf29*, *wtf33*, and *wtf35* in addition to FY29033 *wtf1*, *wtf18*, *wtf35*, and *wtf36* (naming scheme described below). We found that our assemblies matched the Sanger sequencing except at the *Sk wtf2* locus. Our mate-pair sequencing revealed that the Sanger sequencing of *Sk wtf2* missed a Tf transposon and a second *wtf* gene (*wtf34*) in the region, likely due to template switching during polymerase chain reaction amplification. These results suggest that our assembly approach is robust.

We then predicted *wtf* coding sequences based on possible open reading frames and homology to annotated *Sp wtf* genes. Our analyses (discussed below) found additional *wtf* gene variation not represented in the *wtf* genes found in *Sp* or CBS5557.

### Naming *wtf* Genes

There are currently three reported phenotypic classes of intact *wtf* genes: killer meiotic drivers, suppressors of drive, and one essential gene (*Sp wtf21*) (Kim et al. 2010; Hu et al. 2017; Nuckolls et al. 2017; Bravo Núñez, Lange, et al. 2018). It is unknown whether there are other phenotypic classes of *wtf* genes, but it would not be surprising given their vast diversity. To facilitate answering this question and to guide future phenotypic classification of *wtf* genes, we assigned gene names to each *wtf* gene from *Sk*, FY29033, and CBS5557.

For *Sp*, we used existing gene names, and for each other genome, we named genes according to their genomic synteny by comparison with *Sp*. We use *Sk* as an example to explain our naming scheme. At the loci where both *Sk* and *Sp* have one *wtf* gene, we gave the *Sk* gene the same number as *Sp* (e.g., *Sk wtf1*), regardless of sequence identity. For loci where *Sp* has one gene and *Sk* has two genes (e.g., at the *Sp wtf8* locus), we gave the same gene number to the *Sk* gene that was most similar to the *Sp* gene and gave the remaining *Sk wtf* genes increasing numbers (26–35) depending on their order in the *Sk* genome. We followed the same convention for naming the FY29033 and CBS5557 *wtf* genes to facilitate comparisons between isolates (the genes of CBS5557 were already named by Hu et al. [2017] as *cw1*–*cw29*; we provide name translations in supplementary table 1, Supplementary Material online). Supplementary figure 6, Supplementary Material online, shows *wtf* gene names and locations in the four isolates.

### Pervasive Nonallelic Gene Conversion between *wtf* Genes

To examine *wtf* gene evolution, we aligned their coding sequences and generated a maximum-likelihood phylogenetic tree. Naively, we expected that sets of genes from the four sequenced isolates that are found in syntenic loci would group together in well-supported clades on the tree. However, syntenic genes grouped with one another in only a few clades of the tree. The *wtf7*, *wtf11*, *wtf14*, and *wtf15* genes each form well-supported clades that do not include genes from other loci (bootstrap values >95%; supplementary data and supplementary fig. 7, Supplementary Material online). Each of these genes is quite distinct from other *wtf* genes (separated by long branches). The alleles of the *wtf12* and *wtf17* genes also form well-supported clades (>80% support), albeit less diverged from their nearest neighbors (supplementary fig. 7, Supplementary Material online). These genes, however, appear to be losing function in at least some isolates: Shared inactivating mutations in the *wtf12* gene in all four isolates indicate that it pseudogenized prior to the divergence of the isolates, and the *Sp* and *Sk* sequences of *wtf17* also appear pseudogenized.

Despite clear synteny and a very short time (∼2,300 years) since these yeast isolates shared a common ancestor (Jeffares 2018), none of the remaining syntenic *wtf* gene sets forms well-supported clades that exclude *wtf* genes from other loci. Furthermore, there are clear examples of well-supported clades containing genes from different loci. For example, one well-supported clade includes the following genes: *Sk wtf29* and *wtf30*; *Sp wtf19* and *wtf23*; FY29033 *wtf8*, *wtf30*, and *wtf38*; and CBS5557 *wtf29* (highlighted in supplementary fig. 7, Supplementary Material online). Finally, the tree contains two well-supported terminal nodes in which gene pairs at distinct loci from the same isolate (*Sp wtf19* and *wtf23* as well as *FY29033 wtf1* and *wtf35*) form a clade, whereas syntenic genes from other isolates are in distinct clades. These observations are consistent with gene conversion within the *wtf* gene family.

To analyze whether entire *wtf* coding sequences might be overwriting one another by gene conversion, or whether only portions of the genes are involved, we performed GARD (genetic algorithm for recombination detection) analysis on our coding sequence alignment to test for recombination between *wtf* genes (supplementary fig. 8, Supplementary Material online) (Kosakovsky Pond et al. 2006). This algorithm tests the hypothesis that the same phylogenetic tree represents the entire alignment or whether different trees best represent different segments due to recombination. GARD analysis found that the hypothesis of multiple trees best describing different segments was >100 times more likely than the hypothesis of a single tree. In addition, GARD identified three likely segments (*P* < 0.01, supplementary fig. 8, Supplementary Material online). Together, our observations are consistent with widespread nonallelic gene conversion between members of the *wtf* gene family. Such gene conversion obscures the evolutionary history of the *wtf* gene family and means that functional inferences can often not be made across isolates based on shared synteny. This work confirms and expands observations made by Hu et al. (2017) who previously described gene conversion among *Sp* and CBS5557 *wtf* genes. The *wtf7*, *wtf11*, *wtf14*, and *wtf15* genes are likely not participating in this nonallelic gene conversion because the alleles of each of these genes group together in clades in all the trees (supplementary figs. 7 and 8, Supplementary Material online).

**Fig. 2.**
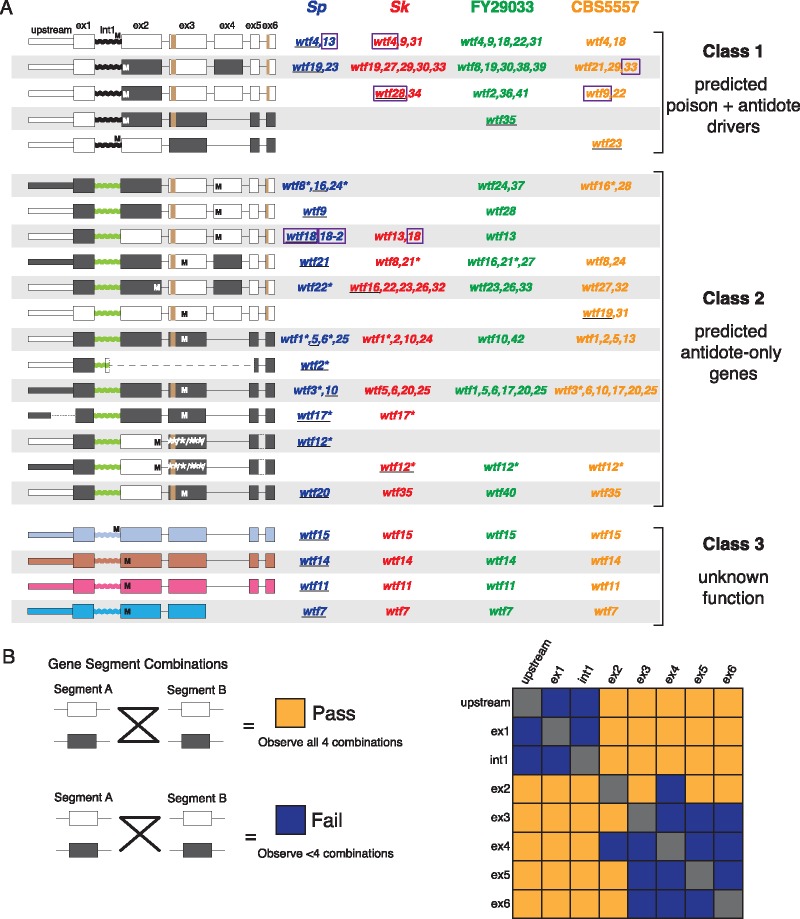
Classification of *wtf* genes based on sequence, and evidence of nonallelic gene conversion. (*A*) Although they are quite diverged from each other, *wtf7*, *wtf11*, *wtf14*, and *wtf15* were placed in a shared class because their sequences are unlike any functionally characterized genes. For the remaining genes, individual gene segments (each exon, intron 1, and the upstream region) from all genes were aligned and classified based on the major clades in maximum likelihood trees (see text for details). Each segment’s clades were color-coded for display purposes (i.e., black/white, black/green coding), and genes were grouped based on gene segment patterns. On the left, we display cartoons of gene structures for each group: boxes indicate exons, “M” indicates in-frame start codons, “/” indicates frameshift mutations, and “*” indicates in-frame stop codons. The repeat regions found in exons 3 and 6 are shown in brown. The names of genes in each class are listed on the right, with the gene illustrated in the cartoon underlined, and pseudogenes denoted with asterisks after gene names. The predicted function of each gene class is shown on the far right. The Class 1 genes are predicted to be meiotic drivers and the Class 2 genes are predicted to be suppressors of drive. Genes with experimentally verified phenotypes have their names outlined with purple boxes. (*B*) Pairwise four-gamete test for recombination (gene conversion) between all pairs of *wtf* gene segments for the genes in Classes 1 and 2. Orange boxes indicate that recombination likely occurred because all four segment combinations were observed. Purple boxes indicate that not all segment combinations were observed.

To further explore *wtf* nonallelic gene conversion, we divided the genes into segments (described below) and compared their evolutionary histories. We excluded *wtf7*, *wtf11*, *wtf14*, and *wtf15* from these analyses because, as described above, they do not appear to have undergone nonallelic gene conversion. Most *wtf* genes have either five or six exons. For ease of comparison, we named the exons 1–6 based on the longest *wtf* genes (fig. 2). The five-exon genes are missing “exon 4,” but the remaining exons can be aligned to those of the six-exon genes (fig. 2). After excluding repetitive regions in exons 3 and 6 (discussed below), we generated alignments and trees (supplementary figs. 9–16 and supplementary data, Supplementary Material online) for each exon separately. We also generated alignments and trees for a conserved region (133–289 bp) upstream of the start codon and for intron 1, regions that in intact *wtf* meiotic drive genes presumably contain the promoters for the antidote and poison transcripts, respectively (Bowen et al. 2003; Nuckolls et al. 2017). The division between segments along intron/exon boundaries was arbitrary: There is no reason that gene conversion should show breakpoints at these boundaries.

Strikingly, trees made from different gene segments do not show the same topology as one another (supplementary figs. 9–16 and supplementary data, Supplementary Material online). This is consistent with nonallelic gene conversion between genes. Although the short length of each segment means that bootstrap support values are generally low throughout the trees, each tree shows a broad subdivision between two main clades of *wtf* genes. For all but the shortest segment (exon 5), these two main clades are separated by a node with high bootstrap support. However, for different gene segments, the two main clades group different subsets of genes together. For example, the first two exons of *Sp* and *Sk wtf9* are very different and are in different main clades. Starting within exon 3, however, the two alleles are much more similar and group in the same main clades (supplementary figs. 10, 11, and 17, Supplementary Material online). One possible explanation for this pattern is that their high similarity in exons 3–6 reflects their original syntenic relationship, but that relationship has been obscured in exons 1 and 2 by gene conversion from another *wtf* gene overwriting sequence in one or both of the isolates.

We used the broad clade divisions defined by the trees for each segment to generate a cartoon representation of this “patchwork” evolutionary history. In the cartoon, each color represents one of the two well-supported clades for each gene segment (fig. 2*A*). We used the color coding to guide grouping the full-length *wtf* genes as shown (fig. 2*A*). We then carried out four-gamete tests to look for evidence of gene conversion between the gene segments (Hudson and Kaplan 1985). Briefly, we considered each of the two major clades for each segment as alternate “alleles.” We then did pairwise comparisons of all gene segments to assay how many of the four possible allele (clade) combinations were observed. The four-gamete test is positive when all four combinations are present; although a simple accumulation of individual sequence changes could explain up to three combinations, the fourth combination can only be explained by recombination (fig. 2*B*). We found that 18/28 comparisons yielded a positive four-gamete test. Although we cannot reconstruct the full history and exact boundaries of gene conversion among *wtf* genes in each isolate, it is clear that the gene family has experienced rampant sequence exchange that could have facilitated rapid functional divergence of the gene family by bringing together new combinations of sequence variants.

Crossing over between syntenic loci likely also contributed to the shuffling of sequence blocks between *wtf* genes. For example, uneven crossover events between loci with multiple distinct *wtf* genes could generate novel hybrid *wtf* genes. Additionally, after a nonallelic gene conversion event has occurred at one locus, allelic recombination at that site can generate additional *wtf* diversity.

### DNA Double-Strand Break Hotspots Are Enriched Near *wtf* Genes

The high level of nonallelic gene conversion among *wtf* genes is surprising because nonallelic homologous recombination (also known as ectopic recombination) is thought to be generally suppressed (Sasaki et al. 2010). This suppression is important because recombination events between nonallelic loci can result in genetic exchanges (crossovers) that cause deleterious chromosome rearrangements (Sasaki et al. 2010). The gene conversion among *wtf* genes we observe could be caused by increased frequency of nonallelic homologous recombination among these genes, or due to selection favoring the products of gene conversion events. The two explanations are not mutually exclusive and both could contribute. The latter idea is difficult to test, so we focused on the first idea. Gene conversion results from the repair of DNA double-strand breaks (DSBs). The initiating DSB could happen near or within the gene converted locus itself, or the break could happen in a different (donor) site that shares homology with the gene converted locus (e.g., another similar *wtf* gene) (Sasaki et al. 2010). DSBs arise at low frequencies in vegetative cells, but are dramatically induced (∼58 breaks per cell in *Sp*) during meiosis (Fowler et al. 2014). Due to their greater numbers and the fact that they have been mapped, we focused our analyses on meiotic DSBs.

Meiotic DSBs do not form randomly and are instead enriched in regions called “hotspots.” *Sp* has 602 DSB hotspots that are generally conserved between *Sp* and *Sk*, so it is reasonable to assume that the *Sp* hotspot map represents the *S. pombe* group (Fowler et al. 2014; Zanders et al. 2014). The *wtf* genes could have elevated gene conversion frequencies if all or a subset of them are near DSB hotspots. A factor known as the “gene conversion tract length” would affect how near to a break *wtf* genes must be in order to be involved in gene conversion events as either donors or recipients. This tract length specifies the amount of DNA that may be incorporated in the DSB repair event and potentially involved in gene conversion. The gene conversion tract length has only been coarsely measured in *Sp* for allelic meiotic recombination at one locus (*ade6*). The observed gene conversion tract lengths were generally <1 kb and occasionally >2 kb (Grimm et al. 1994). It is unknown whether gene conversion varies by locus, and whether tract length is different for allelic repair than for nonallelic recombination. Given this high level of uncertainty, we designated hotspots within 2.5 kb of a *wtf* gene as potential sources of initiating gene conversion events.

We looked for an association between the 602 previously defined *Sp* DSB hotspots and *wtf* loci by calculating the distance between each end of the *wtf* coding sequences and the nearest DSB hotspot (Fowler et al. 2014). There was no DSB hotspot 5′ to the first *wtf* gene on chromosome 3, so we only considered the hotspot 3′ of this coding sequence yielding 47 data points (two ends of each of the 24 loci containing *wtf* genes minus 1). We did the same comparison for all annotated coding sequences (McDowall et al. 2015). We found that DSB hotspots were significantly enriched within 2.5 kb of *wtf* loci as compared with all coding sequences. This enrichment was also significant if we only considered hotspots within 1 kb (table 1, *G*-test *P* < 0.01). Overall, we found that 14 of the 24 *wtf* loci are within 2.5 kb of one or more hotspots.

**Table 1.** DSB Hotspots Are Enriched Near *wtf* Loci.

Distance to DSB	≤2,500 bp	>2,500 bp	≤1,000 bp	>1,000 bp
All CDS	1,764 (17.4%)	8,395 (82.6%)	1,103 (10.9%)	9,056 (89.1%)
*wtf* loci	18 (38.3%)	29 (61.7%)	13 (27.7%)	34 (72.3%)
	*P* < 0.001		*P* = 0.0015	

The genes found within 1 kb of a hotspot include *wtf1*, *wtf9*, *wtf12*, *wtf22*, *wtf25*, and *wtf27*. These genes all show some evidence of participating in nonallelic gene conversion events (i.e., not all alleles of the genes are found on the same row in fig. 2*A*). *wtf7*, *wtf11*, *wtf14*, and *wtf15* are also found within 1 kb of a hotspot, but as discussed above, these genes show no signs of nonallelic gene conversion. This lack of gene conversion is likely because these genes are highly diverged from each other and all other members of the *wtf* gene family (<55% DNA sequence identity).

These analyses suggest that close proximity of some genes to DSB hotspots likely contributes to the high levels of recombination within the *wtf* gene family. Interestingly, we observed no chromosome rearrangements with breakpoints in *wtf* genes in the four isolates with assembled *wtf* genes despite the hotspots and evidence of gene conversion. This suggests that nonallelic homologous recombination events are either preferentially repaired as gene conversions, as opposed to crossovers, or that isolates resulting from such crossovers have been removed by selection because they often generate chromosomes missing essential genes and/or with inviable duplications.

### High Diversity of Intragenic Repeats in *wtf* Proteins

**Fig. 3.**
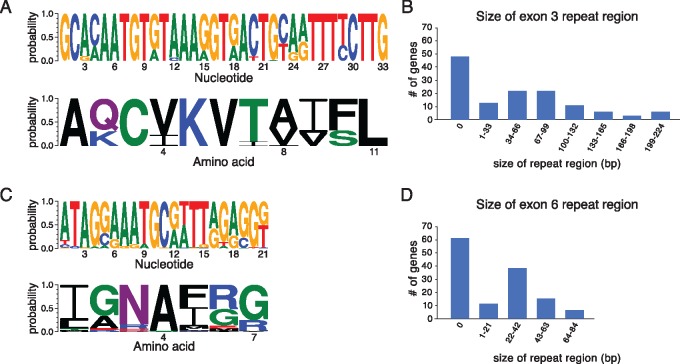
Expansion and contraction of repeat sequences contribute to rapid *wtf* gene evolution. (*A*) DNA (top) and amino acid (bottom) sequence logos representing the repeat region found in exon 3. (*B*) The distribution of exon 3 repeat region size across all assembled *wtf* genes. The sizes are presented in base pairs instead of repeat units because the terminal repeats are not always full length. (*C*) DNA (top) and amino acid (bottom) sequence logos representing the exon 6 repeat region found in many *wtf* genes. (*D*) The distribution of exon 6 repeat sizes in all assembled *wtf* genes.

Insertions and deletions within genes can be an additional source of evolutionary novelty that can result from errors during DNA replication or from recombination (Verstrepen et al. 2005). We looked for evidence of such changes within *wtf* genes and found two repetitive regions that have frequently expanded and contracted during *wtf* evolution. The first of these is a region containing a well-conserved 33-bp repeat sequence near the beginning of exon 3 in most *wtf* genes (fig. 3*A*). Not all of the repeat units are complete. The first repeat is routinely truncated to 21 nucleotides, whereas the last repeat is truncated to between 14 and 26 nucleotides. The *wtf* genes have between 0 and 224 bp of sequence derived from this repeat (fig. 3*B*). A second dynamic repeat region occurs at the start of exon 6 in most genes (fig. 3*C*). This 21 bp repeat unit is less conserved and not all repeat units are complete. This repeat comprises between 0 and 84 bp of sequence in *wtf* genes (fig. 3*D*). Both repeat regions appear unstable in that *wtf* alleles that are otherwise similar can vary in the number of repeat units. For example, the *Sp* and *Sk* alleles of *wtf4* are 93% identical outside of the repeats but have different copy numbers of both repeat segments.

The changes in repeat numbers may be caused by replication slippage or imprecise repair of breaks via allelic homologous recombination (Lovett 2004; Verstrepen et al. 2005). They could also be caused by nonallelic gene conversion with a *wtf* containing a different number of repeats. These changes in repeat numbers may be functionally important because the repeats often overlap predicted transmembrane domains. The function of these repeats is currently unknown, but the number of repeats found in exon 6 can be important for conferring specificity between poison and antidote proteins (Bravo Núñez, Lange, et al. 2018).

### *wtf7*, *wtf11*, and *wtf15* Exhibit High Nonsynonymous Nucleotide Diversity

It is clear that gene duplication, deletion, gene conversion, and changes in repeat units have all acted to generate extensive diversity in the *wtf* gene family. We also wondered whether individual amino acid changes have also played a role in increasing *wtf* diversity. We were limited in the types of molecular evolutionary analyses we could conduct because *S. pombe* does not have sister species that are closely related enough to accurately determine the number of nonsynonymous and synonymous mutations that have occurred along each lineage. The closest relatives to *S. pombe* are *S. octosporus* and *S. cryophilus*, which each share only ∼66% amino acid identity with *S. pombe* (Rhind et al. 2011). We therefore analyzed nonsynonymous (π_N_) and synonymous (π_s_) nucleotide diversity among *S. pombe* isolates (Nei and Li 1979). Ratios of π_N_/π_s_ ≪1 are consistent with a selective disadvantage for variants that change protein sequences. Ratios of π_N_/π_s_ ≫1 are consistent with balancing selection to maintain allele polymorphism or relaxed selective constraint.

Fawcett et al. (2014) previously calculated π genome-wide average for *S. pombe* genes. They found that average π_N_ is 0.00096 whereas π_s_ is 0.006, giving a π_N_/π_s_ ratio of 0.16. We calculated π_N_/π_s_ in the four *wtf* genes that appear not to have participated in nonallelic gene conversion: *wtf7*, *wtf11*, *wtf14*, and *wtf15* (these genes also lack internal repeats that, like gene conversion, could confound analyses). These genes were slightly too short to be included in the analyses of Fawcett et al. (2014), as they only considered genes with >200 nonsynonymous sites.

To calculate π_N_/π_s_, we first assembled the sequences of the four genes from 53 additional *S. pombe* isolates using published 100-bp paired-end read data (Jeffares et al. 2015). This was possible due to the large divergence between each of these genes and all other *wtf* genes. These genes all appear to be single copy in each genome as sequence coverage of these genes was similar to that of single-copy genes on chromosome 3. In many cases, the sequences of orthologous genes were identical between isolates. We found a total of 9 different alleles of *wtf7*, 14 alleles of *wtf11*, 8 alleles of *wtf14*, and 9 alleles of *wtf15*. We found 32, 25, 8, and 19 polymorphisms segregating in these genes, respectively (supplementary table 2, Supplementary Material online). Despite their shorter length, the numerous polymorphisms of at least three of these four genes make it seem unlikely that any unusual π_N_/π_s_ ratios have occurred by chance. We calculated π_N_ and π_s_ using the DNA Sequence Polymorphism (DnaSP) software (Rozas et al. 2017).

We found that all four genes exhibited π_N_/π_s_ ratios greater than the genome average of 0.16 (supplementary table 2, Supplementary Material online). For *wtf7*, we observed a π_N_/π_s_ ratio of 5.7. The π_s_ value for *wtf7* was almost 2-fold lower than the genome-wide average, but π_N_ was nearly 21-fold higher than the genome-wide average, indicating that the high ratio is driven largely by an excess of nonsynonymous polymorphisms. The high ratios for *wtf11* and *wtf15* (0.77 and 1.3) were also largely driven by an increased π_N_, and thus seem likely to indicate balancing selection to maintain multiple protein variants. The high ratio for *wtf14* (0.67), however, was largely driven by a low π_s_.

Although these analyses are limited, they are consistent with the idea that there is not a single fitness optimum for *wtf7*, *wtf11*, and *wtf15* within the *S. pombe* population. Rather, it may be that the fitness of these alleles depends on the context (genetic or environmental) in which they are found.

### Some *wtf* Genes Show Characteristics of Poison-Antidote Systems, Whereas Others May Encode Antidote-Only Suppressors

In addition to facilitating visualization of gene conversion, we grouped the *wtf* genes into the three major classes shown in figure 2 to guide future functional analyses. Briefly, we dubbed the genes that contain in-frame start codons just upstream or near the beginning of exon 2 “Class 1” genes. These exon 2 ATG codons encode the start of Wtf poison protein isoforms and are shared by all of the previously known drivers (fig. 2) (Hu et al. 2017; Nuckolls et al. 2017; Bravo Núñez, Lange, et al. 2018). In addition, we used published long-read RNA sequences to confirm that all the *Sp* Class 1 genes have an alternate transcriptional start site within intron 1 (supplementary fig. 1, Supplementary Material online) that could encode poison transcript isoforms (Kuang et al. 2016). We therefore predict that Class 1 genes are intact meiotic drivers in which transcripts that include all exons encode antidote proteins, and transcripts which exclude exon 1 encode poison proteins.

Most other genes lack both a transcriptional isoform that excludes exon 1 and an in-frame ATG near the start of exon 2: We classify these as Class 2 genes. Due to similarity between the Class 2 genes and the antidote proteins produced by known drivers, we predict that these genes are suppressors of *wtf* drive genes and lack the poison isoform (Lopez Hernandez and Zanders 2018). Indeed, Class 2 contains the only known *wtf* drive suppressor, *Sp wtf18-2* (Bravo Núñez, Lange, et al. 2018). Consistent with the predicted lack of a poison isoform, the *Sk wtf5* and *wtf6* genes do not cause drive in *Sp* (Nuckolls et al. 2017). Notably, we found no *wtf* genes that lack exon 1 that would encode only poison isoforms: It would have been very surprising to find such genes as we predict that they would encode “suicide” alleles unless they were very closely linked to a completely effective suppressor.

Class 3 consists of the remaining genes: *wtf7*, *wtf11*, *wtf14*, and *wtf15*. These genes are diverse and are grouped together only because they all have unknown functions. These genes do have an in-frame start codon near the start of exon 2, like known drivers. However, long-read RNA sequencing data showed no evidence of alternate transcripts for these genes beginning in intron 1 (supplementary fig. 1, Supplementary Material online), so we cannot make a clear prediction about whether they actually encode poison isoforms (Kuang et al. 2016). Furthermore, their increased sequence divergence from the rest of the *wtf* family could suggest divergent functions.

## Discussion

Our study extends previous evolutionary analyses to demonstrate extremely dynamic evolution of the *wtf* gene family in multiple lineages of *S. pombe* (Hu et al. 2017). Although the genomes of different isolates of the *S. pombe* group are nearly identical (>99.5% DNA sequence identity) (Jeffares 2018), the number of *wtf* genes (including pseudogenes) found in the different isolates we studied is variable and the sequences of syntenic genes can be very diverged. This rapid evolution scenario is consistent with molecular arms race models that predict rapid evolution of meiotic drivers and their suppressors (McLaughlin and Malik 2017). It also supports the idea that rapid evolution could be a hallmark of these genes that could be used, along with other features like germline expression and lineage restriction, to facilitate their discovery.

### Rapid Evolution of *wtf* Genes

We observe three mechanisms driving innovation in *wtf* gene sequences. First, as observed by Hu et al. (2017) who previously assayed *Sp* and CBS5557, we found pervasive nonallelic gene conversion affecting most *wtf* genes. We demonstrated that this nonallelic gene conversion was not restricted to a specific portion of the genes and included promoters. The forces driving this gene conversion will require further investigation. It is possible that the *wtf* genes inherently undergo gene conversion at a high rate due to some intrinsic property. For example, the close proximity of a subset of *wtf* loci to meiotic DSB hotspots could facilitate nonallelic recombination within the family. It is also possible that the novel *wtf* sequences generated by gene conversion are frequently advantageous. For example, novel variants could drive or suppress other drivers and thus be maintained by selection.

Second, we found that the number of units of repeat sequences within exons 3 and 6 varies greatly. Such repetitive sequences are known to be unstable and several *wtf* alleles that are otherwise very similar vary in repeat copy number. Although the function of these repeat regions is not clear, the repeats often overlap predicted transmembrane domains, and repeat number can be functionally important. For example, *Sp wtf18* antidote alleles were only able to neutralize *Sp wtf13* poison alleles that had the same number of exon 6 repeats (Bravo Núñez, Lange, et al. 2018). It is possible that the presence of these repeats in *wtf* genes is maintained, at least in part, due to their hypermutability. A high capacity to facilitate rapid gene diversification could be beneficial in genes involved in genetic conflicts.

The third contributor to rapid *wtf* gene evolution we observed in *wtf7*, *wtf11*, and *wtf15* (but not *wtf14*) was an excess of nonsynonymous polymorphisms compared with genome averages (supplementary table 2, Supplementary Material online). Unfortunately, extensive gene conversion limited our analyses to four genes. The *wtf7, wtf11*, and *wtf15* genes have no known functions and are all highly diverged from the experimentally characterized *wtf* genes and each other. The high level of amino acid polymorphism (high π_N_/π_s_) we observed in these genes, however, is consistent with the idea that the fitness of a given allele is context dependent. We have observed this experimentally for *wtf* meiotic drivers and suppressors. Specifically, we observed that the transmission frequency of *wtf13* meiotic drive alleles into viable gametes was different in the presence of a compatible *wtf18* drive suppressor allele (Bravo Núñez, Lange, et al. 2018). We therefore speculate that *wtf7*, *wtf11*, and *wtf15* genes could also be meiotic drivers and/or act as modifiers of meiotic drive.

### Consequences of Rapid Evolution

The rapid evolution of *wtf* genes has led each of the isolates we assayed here to contain a unique suite of *wtf* alleles. The consequences of this *wtf* diversity on *S. pombe* fitness are profound when the organism outcrosses. Although *S. pombe* can grow clonally, it mates and undergoes meiosis to form spores when starved. Although outcrossing occurs in the wild (Fawcett et al. 2014; Farlow et al. 2015; Jeffares et al. 2015; Tusso et al. 2018), determining how often *S. pombe* outcrosses is complicated because the genetic hallmarks of outcrossing are obscured by drive (Lopez Hernandez and Zanders 2018). When nonclonal isolates of *S. pombe* do mate to produce diploids, it is very likely there will be heterozygosity at one or more *wtf* loci. When these diploids undergo meiosis to generate gametes, *wtf* heterozygosity can lead to dramatic loss of fertility due to meiotic drive. This *wtf* heterozygosity is a major cause of the infertility observed in both *Sp*/*Sk* and *Sp*/CBS5557 heterozygous diploids and likely contributes to the generally low fertility of outcrossed (i.e., heterozygous) *S. pombe* diploids (Avelar et al. 2013; Hu et al. 2017; Jeffares et al. 2017; Nuckolls et al. 2017; Lopez Hernandez and Zanders 2018). Driving *wtf* genes are thus limiting the ability of *S. pombe* to enjoy all the fitness benefits of sexual reproduction, perhaps putting this species on a path to extinction.

### Model for *wtf* Family Expansion on Chromosome 3

As noted by Bowen et al. (2003), the introns found in all *wtf* genes argue against gene family expansion by retrotransposition. These authors also suggested that some *wtf* genes coduplicated with their associated LTRs. In other words, *wtf* genes took advantage of the ubiquity of distributed transposon sequences to spread within the genome via nonallelic gene conversion to preexisting LTRs, a process known as segmental duplication (Dennis and Eichler 2016). As most *wtf* loci contain at least one *wtf* gene in the majority of the seven isolates analyzed here, we propose that the segmental duplications of *wtf* genes largely occurred prior to the divergence of these isolates and perhaps the *S. pombe* group.

The exploitation of distributed transposon sequences to facilitate the spread of meiotic drivers may not be specific to *wtf* genes. Transposon sequences are also found near *Spok* genes, a different family of single-gene killer meiotic drivers in the fungus *Podospora anserina*. *Spok* genes are found in as many as 11 copies per genome in some species of fungi (Grognet et al. 2014). Although it is unknown whether *Spok* genes are associated with transposons in other species, segmental duplication to preexisting transposon sequences may have also facilitated growth of the *Spok* gene family.

In addition to segmental duplication, tandem duplications (and deletions) also appear to have contributed to the expansion (and contraction) of the *wtf* gene family. Nonallelic recombination and slippage during DNA replication could be contributing to duplications and deletions. These events appear to have continued after the divergence of the isolates analyzed here because the number of *wtf* genes at any given locus varies (fig. 1*C*). For example, Hu et al. (2017) found that *wtf27*, *wtf33*, and *wtf35* genes were all apparently lost in the *Sp* isolate due to recombination between two LTRs in the same orientation that flanked the genes.

Interestingly, like in the reference genome (*Sp*), the *wtf* genes in all the isolates assayed are highly enriched on what is chromosome 3 in *Sp*. Bowen et al. (2003) proposed that this enrichment in *Sp* could reflect a different evolutionary origin for chromosome 3, suggesting that it was introgressed from a diverged isolate with many *wtf* genes throughout the genome. If this is true, such an introgression event must have preceded the divergence of the isolates analyzed here (fig. 1). We have proposed an alternative hypothesis that the segmental duplication events spreading *wtf* genes occur genome-wide, but that the duplicates on chromosome 3 are preferentially maintained, because *S. pombe* can tolerate aneuploidy of only chromosome 3 and not the other chromosomes (Lopez Hernandez and Zanders 2018). This could be important because when two or more distinct *wtf* drivers compete (i.e., they are linked on opposite haplotypes), nearly all haploid gametes are expected to be destroyed. This was, in fact, observed when CBS5557 *wtf9* and *wtf33* were competed at an allelic locus in *Sp* (Hu et al. 2017). Heterozygous aneuploid or heterozygous diploid gametes, however, inherit both drivers and should be immune to both Wtf poison proteins. *Sp* (and presumably other isolates) only tolerates aneuploidy of chromosome 3, so that the fitness costs of competing drivers could be uniquely offset on chromosome 3 (Lopez Hernandez and Zanders 2018).

It is not clear why antidote-only *wtf* genes that act as suppressors of drive should specifically spread or be maintained on chromosome 3. Loci on this chromosome bear the greatest fitness cost of drivers. This is because loci on chromosome 3 are more likely to be linked in repulsion (i.e., on opposite haplotypes) to drivers that will destroy gametes that inherit them instead of the driver in heterozygous crosses. However, suppressors of drive are predicted to be favored at any unlinked locus because they increase fertility (Crow 1991). It is therefore surprising that antidote-only *wtf* genes have not spread throughout the genome. We favor a model in which the frequent gene conversion among *wtf* genes likely leads to toggling between driving and suppressing *wtf* genes at any given locus. For example, we predict that the *wtf18* gene in FY29033 is a driver, but the *wtf18* alleles in *Sp* are suppressors of drive (fig. 2*A*) (Bravo Núñez, Lange, et al. 2018). This toggling could lead to selective maintenance of *wtf* suppressor loci on chromosome 3 due to the mechanism described above for drivers.

### Lessons for the Design of Gene Drives

The themes we describe for *wtf* gene evolution may be instructive for designing gene drives. Gene drives are engineered drive systems used to control natural populations. The general idea is that natural or artificial drivers can be used to spread traits (e.g., disease resistance) throughout a population or to eliminate a population, for example, by generating extreme sex ratio imbalances (Burt 2014). Analyses of natural drivers and drive suppressors, such as those of the *wtf* family, may prove useful for predicting how engineered gene drives (particularly gamete killers) may evolve if released in natural populations. For example, compact gene drives may duplicate to novel loci within a genome. This risk may be particularly high if the gene drives are integrated next to transposons or other dispersed repetitive elements.

## Materials and Methods

### Yeast Isolates and Whole-Genome Sequencing

The *Sp* (SZY643) and *Sk* (SZY661) strains are described by Nuckolls and Bravo Núñez et al. (Nuckolls et al. 2017). We obtained all other isolates from the National BioResource Center, Japan. We prepared genomic DNA using QIAGEN Genomic-tips (catalog number 10262 and 10243) using the QIAGEN DNA buffer set (catalog number 19060). We followed the kit protocol except that we extended the lyticase treatment to 18 h and the RNase A/Proteinase K treatment to 5 h. The Stowers Institute Molecular Biology core facility prepared the sequencing libraries using the Illumina Nextera Mate-Pair Sample Prep Kit (catalog number FC-132-1001). In total, 5- to 8-kb fragments were selected using a BluePippin machine (Sage Science). The libraries were sequenced (150-bp paired-end reads) on an Illumina MiSeq using the MiSeq Reagent Kit v2 (300 cycle) (catalog number MS-102-2002). Sequence data are available in SRA (accession number PRJNA476416).

### Assaying *wtf* Gene Numbers

We used Geneious version 10.0.7 (https://www.geneious.com; last accessed March 18, 2019) for all sequence analyses, unless otherwise stated, using the “map to reference function” for all short-read alignments. To find *wtf* loci in *Sk*, we identified read pairs from the mate-pair library in which one (or both) reads aligned to a library containing the 25 *Sp wtf* genes (using the default “medium-low sensitivity” aligner setting) (Steps 1 and 2 in fig. 1*A*). The “medium” or “medium-low sensitivity” settings are the suggested settings for next generation sequencing reads. Geneious does not recommend more sensitive settings because “using higher sensitivities is unlikely to improve results and will probably take too long and is usually unnecessary if you have sufficient coverage.” The “medium-low sensitivity” allows 20% mismatched bases, whereas the “medium sensitivity” allows 30%. For the other genomes, we also included the *Sk wtf* genes as reference sequences. From those *wtf*-matching read pairs, we then isolated any “partner” reads that did not align to *wtf* genes by again mapping reads to our reference set of *wtf* genes (“medium sensitivity” setting), this time saving only the individual reads that failed to align to any *wtf* gene (fig. 1*A*, Step 3). We then took these *“wtf*-partner” reads and aligned them to the *Sp* reference genome (“medium sensitivity” setting) (fig. 1*A*, Step 4). This generated pileups of reads flanking *wtf* loci. We inspected the pileups manually to infer the number of *wtf* genes at each locus based on the width and pattern of the pileups, as described in the text. For *Sk* and FY29033, these inferences were confirmed or corrected by assembling the *wtf* loci (see below).

### Assembling *wtf* Genes

To assemble the *wtf* gene(s) at a given locus, we used flanking unique sequences as “bait” to identify all read pairs in the region, and then performed individual de novo assemblies for each *wtf* locus separately. This approach should avoid misassemblies that can occur in whole-genome assemblies at repetitive regions like *wtf* loci. In more detail, we first manually identified coordinates of the sequence pileups described above, adding ∼2-kb flanking sequence (fig. 1*D*, orange bars under the pileups). We excluded LTR sequences and other repetitive DNA sequences from these regions and denote them “orange regions.” We identified all mate-pairs that align to these orange regions (“medium-low sensitivity” setting) (fig. 1*D*, Step 1). We then filtered those reads so that we retained only candidate *wtf* locus reads, and not those from flanking regions. To do this, we defined two additional reference regions flanking the *wtf* locus (“green regions”) that extend the orange region to within ∼500 bp of the *wtf* locus and by ∼15 kb in the other direction (fig. 1*D*, green bars under the pileups). We then aligned the read pairs defined in Step 1 to the green regions (“medium sensitivity” setting), retaining only individual reads that failed to align to the green regions; these reads represent candidate *wtf* locus reads (fig. 1*D*, Steps 2 and 3). Finally, we assembled these candidate *wtf* reads using the Geneious “de novo assemble” function (default “medium sensitivity” setting) (fig. 1*D*, Step 4). We obtained 1–4 contigs in most of these assemblies that we were able to stitch together manually using known *wtf* gene orientations and sequence overlaps.

To validate our approach, we compared the assembled genes to Sanger sequencing of 22 cloned *wtf* genes (Nuckolls et al. 2017; Bravo Núñez, Lange, et al. 2018). For 21 genes, the sequences matched. For the *Sk wtf2* locus, our assemblies detected that our published Sanger sequence of the locus was incorrect due to template switching during polymerase chain reaction (Nuckolls et al. 2017). The frequency of bases that did not match the consensus in the assembled *wtf* loci was similar to that observed in the regions flanking the *wtf* loci, suggesting that the assemblies were not collapsing nonidentical repeated sequences. In addition, we failed to identify polymorphic sites within the assemblies using the default settings in the Geneious SNP caller. Gene sequences and annotations are available in GenBank (accession numbers MH837193–MH837230 and MH837431–MH837459).

### DNA Sequence Alignments, Tree Construction, and Sequence Logos

We aligned DNA sequences of the full-length *wtf* genes (or of *wtf* gene segments) in Geneious using the Geneious aligner with the “global alignment without free end gaps” setting. All alignments are provided as supplementary data, Supplementary Material online. We then generated trees in Geneious using the PHYML plugin (version 2.2.3) with the default settings (HKY85 substitution model, set to optimize tree topology branch length and substitution rate, NNI topology search) with 100 bootstraps. For exons 3 and 6, we aligned only sequences downstream of the repetitive regions found near the beginning of those exons (fig. 3). For *wtf* family-wide gene conversion analysis, we used ran a command-line version of the GARD algorithm (using the general discrete model of site-to-site rate variation with three rate classes) (Kosakovsky Pond et al. 2006). We used Weblogo3 (http://weblogo.threeplusone.com; last accessed March 18, 2019) to generate sequence logos of the repetitive regions (Crooks et al. 2004).

### Analysis of Nucleotide Diversity

We mapped paired-end reads from 54 additional *S. pombe* isolates to the *Sp* reference genome to generate consensus sequences of *wtf7*, *wtf11*, *wtf14*, and *wtf15* in the additional isolates (Jeffares et al. 2015). The assembled sequences of these genes are available in GenBank (accession numbers MH837181–MH837192 and MH837231–MH837430). We then codon-aligned a total of 57 sequences for each gene. We used the GARD algorithm (via the DataMonkey website www.datamonkey.com; last accessed March 18, 2019) to screen each alignment for evidence of gene conversion (using the general discrete model of site-to-site rate variation with three rate classes) (Kosakovsky Pond et al. 2006). GARD did not find evidence for gene conversion in the *wtf7*, *wtf11*, *wtf14*, or *wtf15* alignments. We calculated π_N_/π_s_ using DnaSP (Rozas et al. 2017).

## Supplementary Material

Supplementary data are available at Molecular Biology and Evolution online.

## Supplementary Material

Supplementary_Material_msz052

## References

[msz052-B1] Akera T, Chmatal L, Trimm E, Yang K, Aonbangkhen C, Chenoweth DM, Janke C, Schultz RM, Lampson MA. 2017 Spindle asymmetry drives non-Mendelian chromosome segregation. Science 358 6363: 668–672. 29097549 10.1126/science.aan0092 PMC5906099

[msz052-B2] Avelar AT, Perfeito L, Gordo I, Ferreira MG. 2013 Genome architecture is a selectable trait that can be maintained by antagonistic pleiotropy. Nat Commun. 4:2235. 23974178 10.1038/ncomms3235

[msz052-B3] Bowen NJ, Jordan IK, Epstein JA, Wood V, Levin HL. 2003 Retrotransposons and their recognition of pol II promoters: a comprehensive survey of the transposable elements from the complete genome sequence of *Schizosaccharomyces pombe*. Genome Res. 13 9: 1984–1997. 12952871 10.1101/gr.1191603 PMC403668

[msz052-B4] Bravo Núñez MA, Lange JJ, Zanders SE. 2018 A suppressor of a wtf poison-antidote meiotic driver acts via mimicry of the driver's antidote. PLoS Genet. 14 11: e1007836. 30475921 10.1371/journal.pgen.1007836 PMC6283613

[msz052-B5] Bravo Núñez MA, Nuckolls NL, Zanders SE. 2018 Genetic villains: killer meiotic drivers. Trends Genet. 34 6: 424–433. 29499907 10.1016/j.tig.2018.02.003 PMC5959745

[msz052-B6] Burt A. 2014 Heritable strategies for controlling insect vectors of disease. Philos Trans R Soc Lond B Biol Sci. 369 1645: 20130432. 24821918 10.1098/rstb.2013.0432 PMC4024225

[msz052-B7] Burt A, Trivers R. 2006 Genes in conflict: the biology of selfish genetic elements. Cambridge: Belknap Press of Harvard University Press.

[msz052-B8] Cocquet J, Ellis PJ, Mahadevaiah SK, Affara NA, Vaiman D, Burgoyne PS. 2012 A genetic basis for a postmeiotic X versus Y chromosome intragenomic conflict in the mouse. PLoS Genet. 8 9: e1002900. 23028340 10.1371/journal.pgen.1002900 PMC3441658

[msz052-B9] Crooks GE, Hon G, Chandonia JM, Brenner SE. 2004 WebLogo: a sequence logo generator. Genome Res. 14 6: 1188–1190. 15173120 10.1101/gr.849004 PMC419797

[msz052-B10] Crow JF. 1991 Why is Mendelian segregation so exact? Bioessays 13 6: 305–312. 1909864 10.1002/bies.950130609

[msz052-B11] Daugherty MD, Malik HS. 2012 Rules of engagement: molecular insights from host-virus arms races. Annu Rev Genet. 46:677–700. 23145935 10.1146/annurev-genet-110711-155522

[msz052-B12] Dawe RK, Lowry EG, Gent JI, Stitzer MC, Swentowsky KW, Higgins DM, Ross-Ibarra J, Wallace JG, Kanizay LB, Alabady M, et al 2018 A kinesin-14 motor activates neocentromeres to promote meiotic drive in maize. Cell 173 4: 839–850.e18. 29628142 10.1016/j.cell.2018.03.009

[msz052-B13] Dennis MY, Eichler EE. 2016 Human adaptation and evolution by segmental duplication. Curr Opin Genet Dev. 41:44–52. 27584858 10.1016/j.gde.2016.08.001 PMC5161654

[msz052-B14] Didion JP, Morgan AP, Clayshulte AM, McMullan RC, Yadgary L, Petkov PM, Bell TA, Gatti DM, Crowley JJ, Hua K, et al 2015 A multi-megabase copy number gain causes maternal transmission ratio distortion on mouse chromosome 2. PLoS Genet. 11 2: e1004850. 25679959 10.1371/journal.pgen.1004850 PMC4334553

[msz052-B15] Farlow A, Long H, Arnoux S, Sung W, Doak TG, Nordborg M, Lynch M. 2015 The spontaneous mutation rate in the fission yeast *Schizosaccharomyces pombe*. Genetics 201 2: 737–744. 26265703 10.1534/genetics.115.177329 PMC4596680

[msz052-B16] Fawcett JA, Iida T, Takuno S, Sugino RP, Kado T, Kugou K, Mura S, Kobayashi T, Ohta K, Nakayama J, et al 2014 Population genomics of the fission yeast *Schizosaccharomyces pombe*. PLoS One 9 8: e104241. 25111393 10.1371/journal.pone.0104241 PMC4128662

[msz052-B17] Fishman L, Saunders A. 2008 Centromere-associated female meiotic drive entails male fitness costs in monkeyflowers. Science 322 5907: 1559–1562. 19056989 10.1126/science.1161406

[msz052-B18] Fowler KR, Sasaki M, Milman N, Keeney S, Smith GR. 2014 Evolutionarily diverse determinants of meiotic DNA break and recombination landscapes across the genome. Genome Res. 24 10: 1650–1664. 25024163 10.1101/gr.172122.114 PMC4199369

[msz052-B19] Grimm C, Bahler J, Kohli J. 1994 M26 recombinational hotspot and physical conversion tract analysis in the ade6 gene of *Schizosaccharomyces pombe*. Genetics 136 1: 41–51. 7908005 10.1093/genetics/136.1.41 PMC1205790

[msz052-B20] Grognet P, Lalucque H, Malagnac F, Silar P. 2014 Genes that bias Mendelian segregation. PLoS Genet. 10 5: e1004387. 24830502 10.1371/journal.pgen.1004387 PMC4022471

[msz052-B21] Helleu Q, Gerard PR, Dubruille R, Ogereau D, Prud'homme B, Loppin B, Montchamp-Moreau C. 2016 Rapid evolution of a Y-chromosome heterochromatin protein underlies sex chromosome meiotic drive. Proc Natl Acad Sci U S A. 113 15: 4110–4115. 26979956 10.1073/pnas.1519332113 PMC4839453

[msz052-B22] Hu W, Jiang ZD, Suo F, Zheng JX, He WZ, Du LL. 2017 A large gene family in fission yeast encodes spore killers that subvert Mendel's law. Elife 6:e26057. 10.7554/eLife.26057 PMC5478263 28631610

[msz052-B23] Hu W, Suo F, Du LL. 2015 Bulk segregant analysis reveals the genetic basis of a natural trait variation in fission yeast. Genome Biol Evol. 7 12: 3496–3510. 26615217 10.1093/gbe/evv238 PMC4700965

[msz052-B24] Hudson RR, Kaplan NL. 1985 Statistical properties of the number of recombination events in the history of a sample of DNA sequences. Genetics 111 1: 147–164. 4029609 10.1093/genetics/111.1.147 PMC1202594

[msz052-B25] Jeffares DC. 2018 The natural diversity and ecology of fission yeast. Yeast 35 3: 253–260. 29084364 10.1002/yea.3293

[msz052-B26] Jeffares DC, Jolly C, Hoti M, Speed D, Shaw L, Rallis C, Balloux F, Dessimoz C, Bahler J, Sedlazeck FJ. 2017 Transient structural variations have strong effects on quantitative traits and reproductive isolation in fission yeast. Nat Commun. 8:14061. 28117401 10.1038/ncomms14061 PMC5286201

[msz052-B27] Jeffares DC, Rallis C, Rieux A, Speed D, Převorovský M, Mourier T, Marsellach FX, Iqbal Z, Lau W, Cheng TMK, et al 2015 The genomic and phenotypic diversity of *Schizosaccharomyces pombe*. Nat Genet. 47 3: 235–241. 25665008 10.1038/ng.3215 PMC4645456

[msz052-B28] Kim DU, Hayles J, Kim D, Wood V, Park HO, Won M, Yoo HS, Duhig T, Nam M, Palmer G, et al 2010 Analysis of a genome-wide set of gene deletions in the fission yeast *Schizosaccharomyces pombe*. Nat Biotechnol. 28 6: 617–623. 20473289 10.1038/nbt.1628 PMC3962850

[msz052-B29] Kosakovsky Pond SL, Posada D, Gravenor MB, Woelk CH, Frost SD. 2006 Automated phylogenetic detection of recombination using a genetic algorithm. Mol Biol Evol. 23 10: 1891–1901. 16818476 10.1093/molbev/msl051

[msz052-B30] Kuang Z, Boeke JD, Canzar S. 2016 The dynamic landscape of fission yeast meiosis alternative-splice isoforms. Genome Res. 27(1):145–156. 10.1101/gr.208041.116 PMC5204338 27856494

[msz052-B31] Lin CJ, Hu F, Dubruille R, Vedanayagam J, Wen J, Smibert P, Loppin B, Lai EC. 2018 The hpRNA/RNAi pathway is essential to resolve intragenomic conflict in the *Drosophila* male germline. Dev Cell. 46 3: 316–326.e315. 30086302 10.1016/j.devcel.2018.07.004 PMC6114144

[msz052-B32] Lindholm AK, Dyer KA, Firman RC, Fishman L, Forstmeier W, Holman L, Johannesson H, Knief U, Kokko H, Larracuente AM, et al 2016 The ecology and evolutionary dynamics of meiotic drive. Trends Ecol Evol. 31 4: 315–326. 26920473 10.1016/j.tree.2016.02.001

[msz052-B33] Lopez Hernandez JF, Zanders SE. 2018 Veni, vidi, vici: the success of wtf meiotic drivers in fission yeast. Yeast 35(7):447–453. 10.1002/yea.3305 PMC6033644 29322557

[msz052-B34] Lovett ST. 2004 Encoded errors: mutations and rearrangements mediated by misalignment at repetitive DNA sequences. Mol Microbiol. 52 5: 1243–1253. 15165229 10.1111/j.1365-2958.2004.04076.x

[msz052-B35] McDowall MD, Harris MA, Lock A, Rutherford K, Staines DM, Bahler J, Kersey PJ, Oliver SG, Wood V. 2015 PomBase 2015: updates to the fission yeast database. Nucleic Acids Res. 43(Database issue): D656–D661. 25361970 10.1093/nar/gku1040 PMC4383888

[msz052-B36] McLaughlin RN Jr, Malik HS. 2017 Genetic conflicts: the usual suspects and beyond. J Exp Biol. 220(Pt 1): 6–17. 28057823 10.1242/jeb.148148 PMC5278622

[msz052-B37] Nei M, Li WH. 1979 Mathematical model for studying genetic variation in terms of restriction endonucleases. Proc Natl Acad Sci U S A. 76 10: 5269–5273. 291943 10.1073/pnas.76.10.5269 PMC413122

[msz052-B38] Nuckolls NL, Bravo Núñez MA, Eickbush MT, Young JM, Lange JJ, Yu JS, Smith GR, Jaspersen SL, Malik HS, Zanders SE. 2017 wtf genes are prolific dual poison-antidote meiotic drivers. Elife 6:e26033. 10.7554/eLife.26033 PMC5478261 28631612

[msz052-B39] Phadnis N, Orr HA. 2009 A single gene causes both male sterility and segregation distortion in *Drosophila* hybrids. Science 323 5912: 376–379. 19074311 10.1126/science.1163934 PMC2628965

[msz052-B40] Rhind N, Chen Z, Yassour M, Thompson DA, Haas BJ, Habib N, Wapinski I, Roy S, Lin MF, Heiman DI, et al 2011 Comparative functional genomics of the fission yeasts. Science 332 6032: 930–936. 21511999 10.1126/science.1203357 PMC3131103

[msz052-B41] Rozas J, Ferrer-Mata A, Sanchez-DelBarrio JC, Guirao-Rico S, Librado P, Ramos-Onsins SE, Sanchez-Gracia A. 2017 DnaSP 6: DNA sequence polymorphism analysis of large data sets. Mol Biol Evol. 34 12: 3299–3302. 29029172 10.1093/molbev/msx248

[msz052-B42] Sasaki M, Lange J, Keeney S. 2010 Genome destabilization by homologous recombination in the germ line. Nat Rev Mol Cell Biol. 11 3: 182–195. 20164840 10.1038/nrm2849 PMC3073813

[msz052-B43] Shen R, Wang L, Liu X, Wu J, Jin W, Zhao X, Xie X, Zhu Q, Tang H, Li Q, et al 2017 Genomic structural variation-mediated allelic suppression causes hybrid male sterility in rice. Nat Commun. 8 1: 1310. 29101356 10.1038/s41467-017-01400-y PMC5670240

[msz052-B44] Singh G, Klar AJ. 2002 The 2.1-kb inverted repeat DNA sequences flank the mat2, 3 silent region in two species of *Schizosaccharomyces* and are involved in epigenetic silencing in *Schizosaccharomyces pombe*. Genetics 162 2: 591–602. 12399374 10.1093/genetics/162.2.591 PMC1462298

[msz052-B45] Tao Y, Araripe L, Kingan SB, Ke Y, Xiao H, Hartl DL. 2007 A sex-ratio meiotic drive system in *Drosophila simulans*. II: an X-linked distorter. PLoS Biol. 5 11: e293. 17988173 10.1371/journal.pbio.0050293 PMC2062476

[msz052-B46] Tao Y, Masly JP, Araripe L, Ke Y, Hartl DL. 2007 A sex-ratio meiotic drive system in *Drosophila simulans*. I: An autosomal suppressor. PLoS Biol. 5 11: e292. 17988172 10.1371/journal.pbio.0050292 PMC2062475

[msz052-B47] Tusso S, Nieuwenhuis BPS, Sedlazeck FJ, Davey JW, Jeffares D, Wolf JBW. 2018 Ancestral admixture is the main determinant of global biodiversity in fission yeast. bioRxiv. https://doi.org/10.1101/415091 10.1093/molbev/msz126 PMC6736153 31225876

[msz052-B48] Verstrepen KJ, Jansen A, Lewitter F, Fink GR. 2005 Intragenic tandem repeats generate functional variability. Nat Genet. 37 9: 986–990. 16086015 10.1038/ng1618 PMC1462868

[msz052-B49] Wood V, Gwilliam R, Rajandream MA, Lyne M, Lyne R, Stewart A, Sgouros J, Peat N, Hayles J, Baker S, et al 2002 The genome sequence of *Schizosaccharomyces pombe*. Nature 415 6874: 871–880. 11859360 10.1038/nature724

[msz052-B50] Wood V, Harris MA, McDowall MD, Rutherford K, Vaughan BW, Staines DM, Aslett M, Lock A, Bahler J, Kersey PJ, et al 2012 PomBase: a comprehensive online resource for fission yeast. Nucleic Acids Res. 40(Database issue): D695–D699. 22039153 10.1093/nar/gkr853 PMC3245111

[msz052-B51] Xie Y, Xu P, Huang J, Ma S, Xie X, Tao D, Chen L, Liu YG. 2017 Interspecific hybrid sterility in rice is mediated by OgTPR1 at the S1 locus encoding a peptidase-like protein. Mol Plant. 10 8: 1137–1140. 28533193 10.1016/j.molp.2017.05.005

[msz052-B52] Yu X, Zhao Z, Zheng X, Zhou J, Kong W, Wang P, Bai W, Zheng H, Zhang H, Li J, et al 2018 A selfish genetic element confers non-Mendelian inheritance in rice. Science 360 6393: 1130–1132. 29880691 10.1126/science.aar4279

[msz052-B53] Zanders SE, Eickbush MT, Yu JS, Kang JW, Fowler KR, Smith GR, Malik HS. 2014 Genome rearrangements and pervasive meiotic drive cause hybrid infertility in fission yeast. Elife 3:e02630. 24963140 10.7554/eLife.02630 PMC4066438

