# Supplementary material for: Killer Meiotic Drive and Dynamic Evolution of the wtf Gene Family
Source: Mol Biol Evol. 2019 Apr 16;36(6):1201–14. doi: 10.1093/molbev/msz052 (PMC6526906; doi:10.1093/molbev/msz052)
Supplement: Supplementary_Material_msz052 [file supplementary_material_msz052.zip › Supplemental legends.docx]

**Supplemental Files:** Contain the alignments described in the paper in both .txt and .fasta formats.

**Supplemental Table 1:** The names previously given to each CBS5557 *wtf* gene by Hu et al. are shown on the left and the names we use in this work are shown on the right.

**Supplemental Table 2:**  Analysis of DNA sequence polymorphisms amongst 57 isolates of *S. pombe* for the *wtf7*, *wtf11*, *wtf14* and *wtf15* genes. The genome-wide averages of π_N_, π_s_, and π_N_/π_S_ from Fawcett et al. (Fawcett, et al. 2014), are also shown.

­**Supplemental Figure 1**: Representative long RNA sequence reads (dark grey) from Kuang et al. (Kuang, et al. 2016) are shown aligned to *Sp wtf* genes. The solid lines represent UTRs, boxes are exons and the thin lines are introns (i.e. they were not found in the reads). The blue gene annotations are from PomBase. The light grey annotations are based on the long read RNA sequencing reads. The light grey annotations were used for the coding sequences in this work. Predicted pseudogenes have an * after the gene name. On the annotations, the light grey annotations used in this work, the ‘*’s indicate in frame stop codons and the ‘/’s indicate frameshift mutations.

**Supplemental Figure 2:** Insert sizes of mate-pair libraries. Most pairs of reads map as expected for mate-pair fragments (i.e. the 3’ end of the reads point away from each other in the genome) with insert size ~ 6-10 kb. These were the reads that were useful in identifying and assembling *wtf* loci. However, the mate-pair library prep is not 100% efficient, and also generates a subset of reads with inserts of < 1 kb that typically map as regular paired-end reads (i.e. the 3’ end of the reads point towards each other in the genome); these reads were generally discarded by the selective steps of our sequence analysis pipelines (Figure 1A and 1D).

**Supplemental Figure 3:** The width of the sequence pileups used to identify loci containing *wtf* genes and to predict the number of *wtf* genes at each locus. The width of both the 5’ and 3’ pileups is shown for loci with one, two, or three verified *wtf* genes from *Sp* (blue), *Sk* (red), and FY29033 (green). The widest pileup at a given locus was used to predict the number of *wtf* genes when the pileups were asymmetric (e.g. in the loci with three *wtf* genes).

**Supplemental Figure 4:** DNA sequence read pileups flanking *wtf* loci for atypical loci, showing representatives of the patterns not shown in Figure 1B. In general, the atypical pileup patterns were caused by Tf transposon insertions in the sequenced isolate that were not present in the *Sp* reference genome to which the reads were aligned. These transposon insertions make the actual genome from which the reads were derived different from the reference genome and unique sequences (not transposons) are needed next to a *wtf* locus to form a pileup. The Tf insertions were discovered during assembly of the loci. Black arrows indicate the locations of *wtf* genes and green arrows represent Tf transposons.

**Supplemental Figure 5:** Sequence coverage of the *wtf23* region in FY29030 and the *wtf33* region in FY28989. We infer from the roughly doubled coverage of those regions that they are duplicated in those isolates. In addition, high sequence identity across the duplicated regions within each isolate are consistent with recent duplications and very little divergence between the two copies.

**Supplemental Figure 6:** The *wtf* gene names are shown mapped onto the karyotype of *Sp*, although not all the isolates share this karyotype. Genes on the Watson strand are shown above each chromosome, whereas genes on the Crick strand are shown below chromosomes. Experimentally confirmed drivers and genes we predict to be intact drivers (Class 1 in Figure 2) are shown in purple, predicted suppressors (Class 2 in Figure 2) are shown in black and the genes with unknown functions (Class 3 in Figure 2) are shown in light blue text. Predicted pseudogenes are indicated with an asterisk.

**Supplemental Figure 7:** Maximum likelihood tree generated by PhyML (executed in Geneious) including full-length ORF sequences of all *wtf* genes from *Sp*, *Sk*, FY29033 and CBS5557 (alignment length 1,465 bp). The tree is unrooted, but is shown with arbitrary rooting in (A) to facilitate reading the branch labels. Predicted pseudogenes are indicated with an *. Nodes with ≥95% bootstrap support are indicated with red circles. The same tree is shown unrooted in (B). The *wtf7* (dark blue), *wtf11* (pink), *wtf14* (brown) and *wtf15* (light blue) clades are each highlighted. The clade highlighted in green was discussed in the text as an example. The scale bar indicates nucleotide substitutions per site.

**Supplemental Figure 8:** GARD analysis of all *wtf* ORF sequences from *Sp*, *Sk*, FY29033 and CBS5557. This analysis found that a hypothesis allowing multiple trees for different segments of the alignment is >100 times more likely than a hypothesis allowing only a single tree, supporting that recombination operates within *wtf* genes. The analysis identified two likely breakpoints corresponding to positions 615 and 1047 in the alignment, yielding three segments as depicted by the colored rectangles at the top of the figure. Both breakpoints have strong statistical support (***; p<0.01). The trees generated for each segment (below) are distinct.

**Supplemental figure 9:** Maximum likelihood tree for exon 1 of the *wtf* genes (alignment length 150 bp). See legend to Supplemental Figure 7 for details. The grey shaded box corresponds to the black color coded exon in the cartoons in Figure 2A.

**Supplemental figure 10:** Maximum likelihood tree for exon 2 of the *wtf* genes (alignment length 381 bp). See legend to Supplemental Figure 7 for details. The grey shaded box corresponds to the black color coded exon in the cartoons in Figure 2A.

**Supplemental figure 11:** Maximum likelihood tree for exon 3, excluding repeats, of the *wtf* genes (alignment length 203 bp). See legend to Supplemental Figure 7 for details. The grey shaded box corresponds to the black color coded exon in the cartoons in Figure 2A.

**Supplemental figure 12:** Maximum likelihood tree for exon 4 of the 6-exon *wtf* genes (alignment length 192 bp). See legend to Supplemental Figure 7 for details. The grey shaded box corresponds to the black color coded exon in the cartoons in Figure 2A.

**Supplemental figure 13:** Maximum likelihood tree for exon 5 from the 6-exon *wtf* genes and the homologous exon 4 of the 5-exon *wtf* genes (alignment length 65 bp). See legend to Supplemental Figure 7 for details. The grey shaded box corresponds to the black color coded exon in the cartoons in Figure 2A.

**Supplemental figure 14:** Maximum likelihood tree for exon 6, excluding repeats, from the 6-exon *wtf* genes and the homologous exon 5 of the 5-exon *wtf* genes (alignment length 69 bp). See legend to Supplemental Figure 7 for details. The grey shaded box corresponds to the black color coded exon in the cartoons in Figure 2A.

**Supplemental figure 15:** Maximum likelihood tree of the region upstream of *wtf* genes (alignment length 303 bp). See legend to Supplemental Figure 7 for details. The grey shaded box corresponds to the black color coded region in the cartoons in Figure 2A.

**Supplemental figure 16:** Maximum likelihood tree for intron 1 of the *wtf* genes (alignment length 280 bp). See legend to Supplemental Figure 7 for details. The grey shaded box corresponds to the green color coded intron in the cartoons in Figure 2A.

**Supplemental Figure 17:** A codon alignment of the *wtf9* genes from *Sp* and *Sk*. Purple boxes represent exons; all DNA and amino acid sequence variants are highlighted.
