# Supplementary material for: Killer Meiotic Drive and Dynamic Evolution of the wtf Gene Family
Source: Mol Biol Evol. 2019 Apr 16;36(6):1201–14. doi: 10.1093/molbev/msz052 (PMC6526906; doi:10.1093/molbev/msz052)
Supplement: Supplementary_Material_msz052 [file supplementary_material_msz052.zip › Supplemental Table 1 MTEv2.pdf]

Supplemental Table 1

CBS5557 gene names	
**Hu et al.**	**this work**
cw1	*wtf1*
cw2	*wtf2*
cw3	*wtf3*
cw4	*wtf4*
cw5	*wtf5*
cw6a	*wtf6*
cw6b	*wtf28*
cw7	*wtf7*
cw8a	*wtf29*
cw8b	*wtf8*
cw9	*wtf9*
cw10a	*wtf31*
cw10b	*wtf10*
cw11	*wtf11*
cw11x	*wtf32*
cw12	*wtf12*
cw13	*wtf13*
cw14	*wtf14*
cw15	*wtf15*
cw16	*wtf16*
cw17	*wtf17*
cw18	*wtf18*
cw19	*wtf19*
cw20	*wtf20*
cw21	*wtf21*
cw22	*wtf22*
cw23	*wtf23*
cw24	*wtf24*
cw25	*wtf25*
cw26	*wtf35*
cw27	*wtf33*
cw28	*wtf27*
