# Supplementary material for: Killer Meiotic Drive and Dynamic Evolution of the wtf Gene Family
Source: Mol Biol Evol. 2019 Apr 16;36(6):1201–14. doi: 10.1093/molbev/msz052 (PMC6526906; doi:10.1093/molbev/msz052)
Supplement: Supplementary_Material_msz052 [file supplementary_material_msz052.zip › Supplemental Table 2 MTEv2.pdf]

Supplemental Table 2

	gene				genomic average (45)
	wtf7	*wtf11*	*wtf14*	*wtf15*	
**number of sequences**	57	57	57	57	
**unique sequences**	9	14	8	9	
**number of base pairs**	654	765	687	765	
**number of codons**	218	255	229	255	
**non-synonymous polymorphisms**	28	17	6	15	
**synonymous polymorphisms**	4	8	2	4	
**N (non-synon sites)**	508.28	599.8	529.24	592.95	
**S (synon sites)**	145.72	165.2	157.76	172.05	
**π (raw)**	1.61E-02	7.76E-03	1.75E-03	5.92E-03	2.70E-03
**π (Jukes-Cantor)**	1.64E-02	7.82E-03	1.76E-03	5.98E-03	
**π-N (raw)**	1.97E-02	7.30E-03	1.57E-03	6.22E-03	9.60E-04
**π-S (raw)**	3.47E-03	9.42E-03	2.36E-03	4.90E-03	6.00E-03
**π-N/π-S (raw)**	**5.68**	**0.77**	**0.67**	**1.27**	**0.16**
**π-N (Jukes-Cantor)**	2.02E-02	7.36E-03	1.57E-03	6.29E-03	
**π-S (Jukes-Cantor)**	3.49E-03	9.52E-03	2.38E-03	4.94E-03	
**π-N/π-S (Jukes-Cantor)**	5.77	0.77	0.66	1.27	
