# Supplementary figures and images for: Killer Meiotic Drive and Dynamic Evolution of the wtf Gene Family
Source: Mol Biol Evol. 2019 Apr 16;36(6):1201–14. doi: 10.1093/molbev/msz052 (PMC6526906; doi:10.1093/molbev/msz052)

# Supplemental Figure 1 (page 1 of 3)

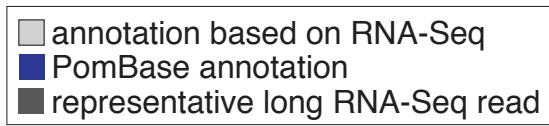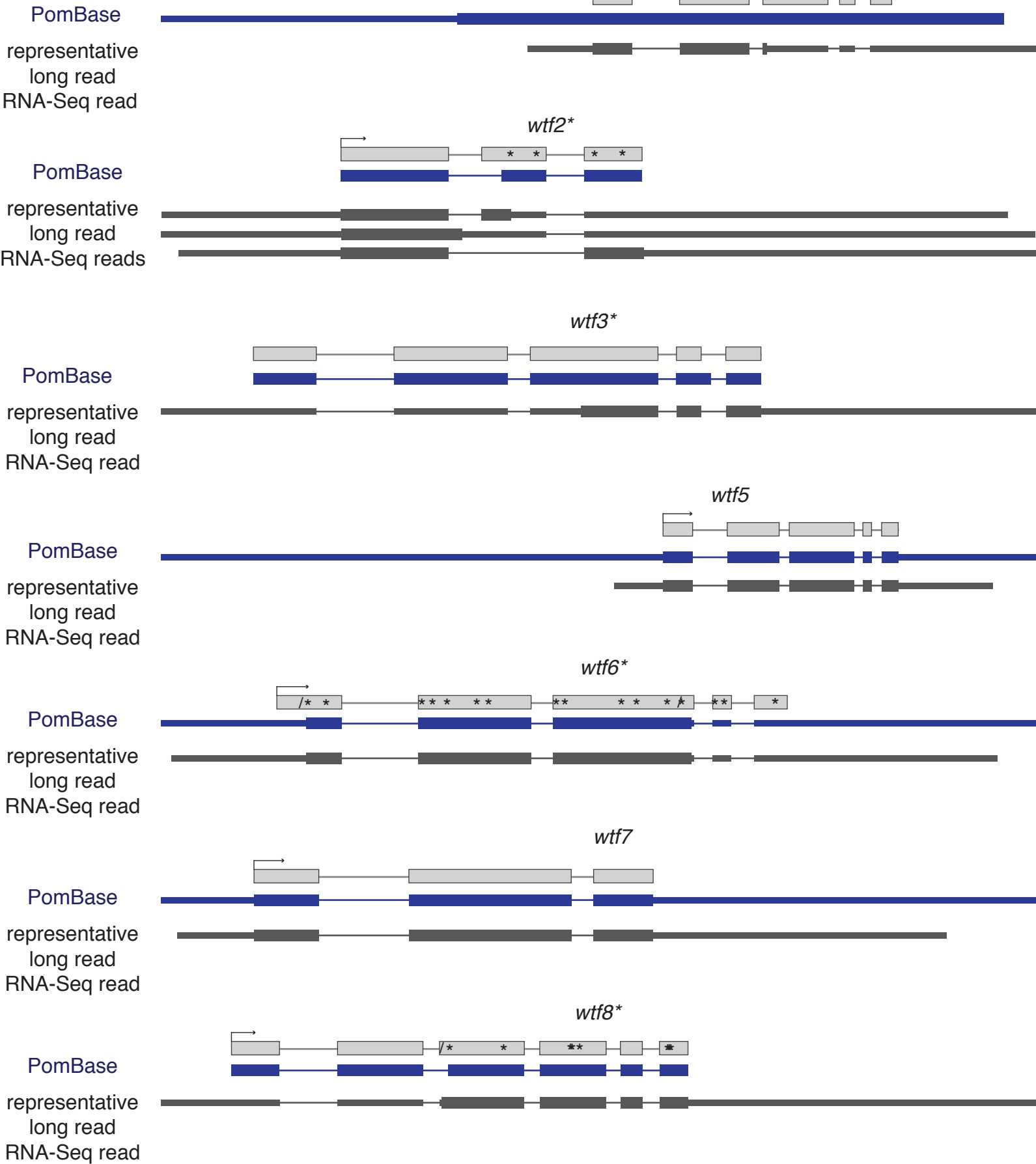

Supplement: Supplementary_Material_msz052 [file supplementary_material_msz052.zip › Supplemental Figure 1 Page 1 MTEv7.pdf]

# Supplemental Figure 1 (page 2 of 3)

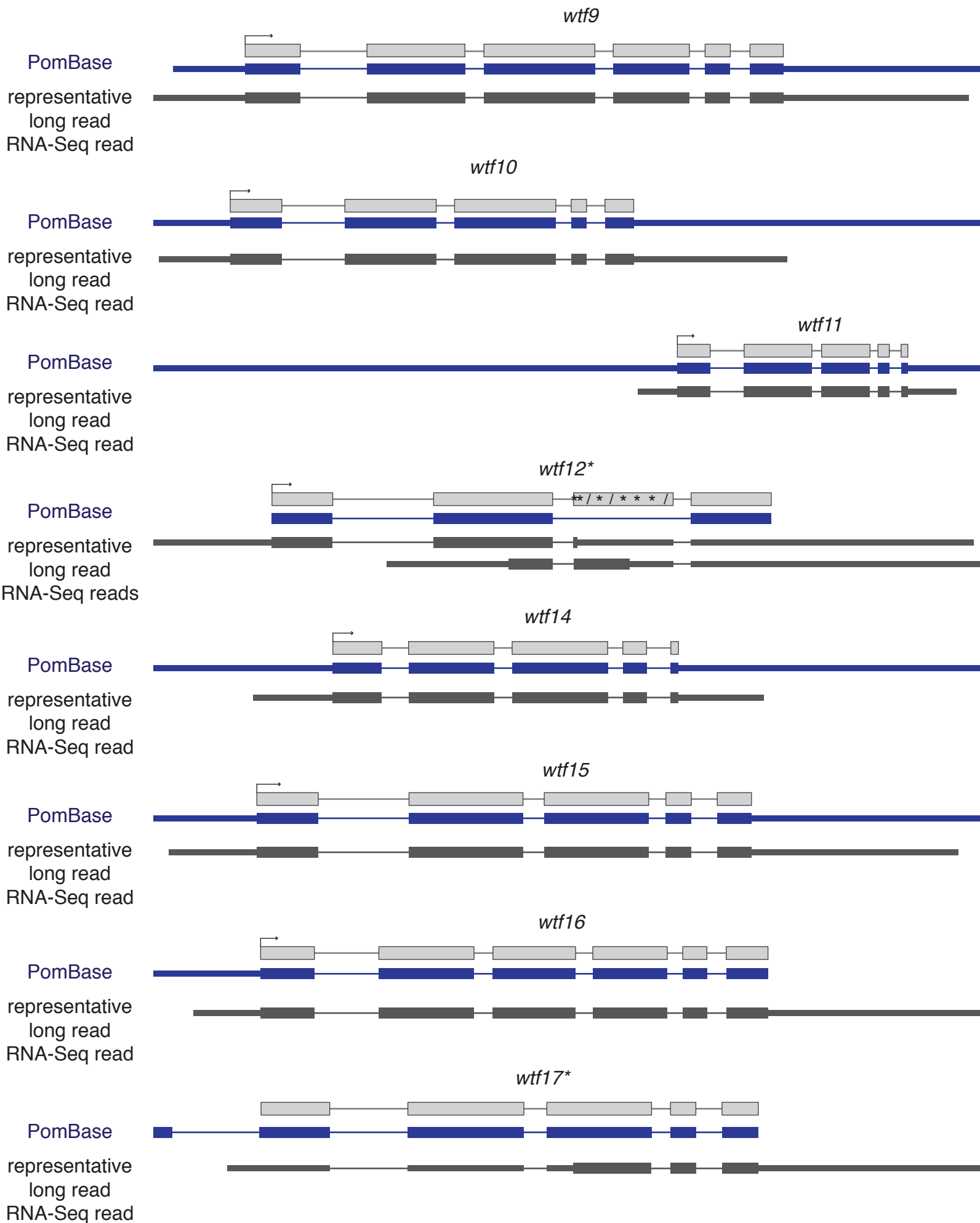

Supplement: Supplementary_Material_msz052 [file supplementary_material_msz052.zip › Supplemental Figure 1 Page 2 MTEv7.pdf]

# Supplemental Figure 1 (page 3 of 3)

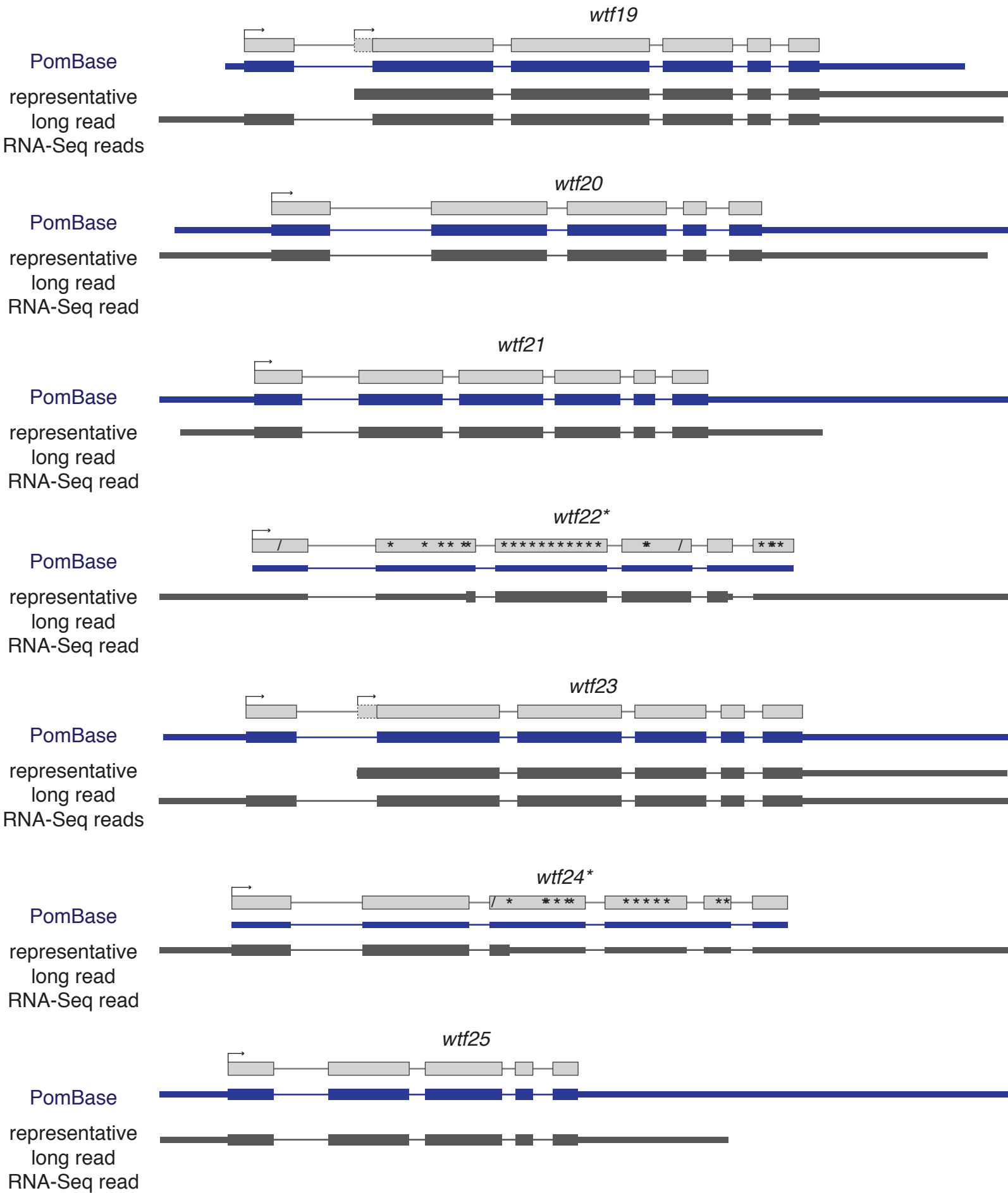

Supplement: Supplementary_Material_msz052 [file supplementary_material_msz052.zip › Supplemental Figure 1 Page 3 MTEv6.pdf]

Supplemental Figure 10

A)

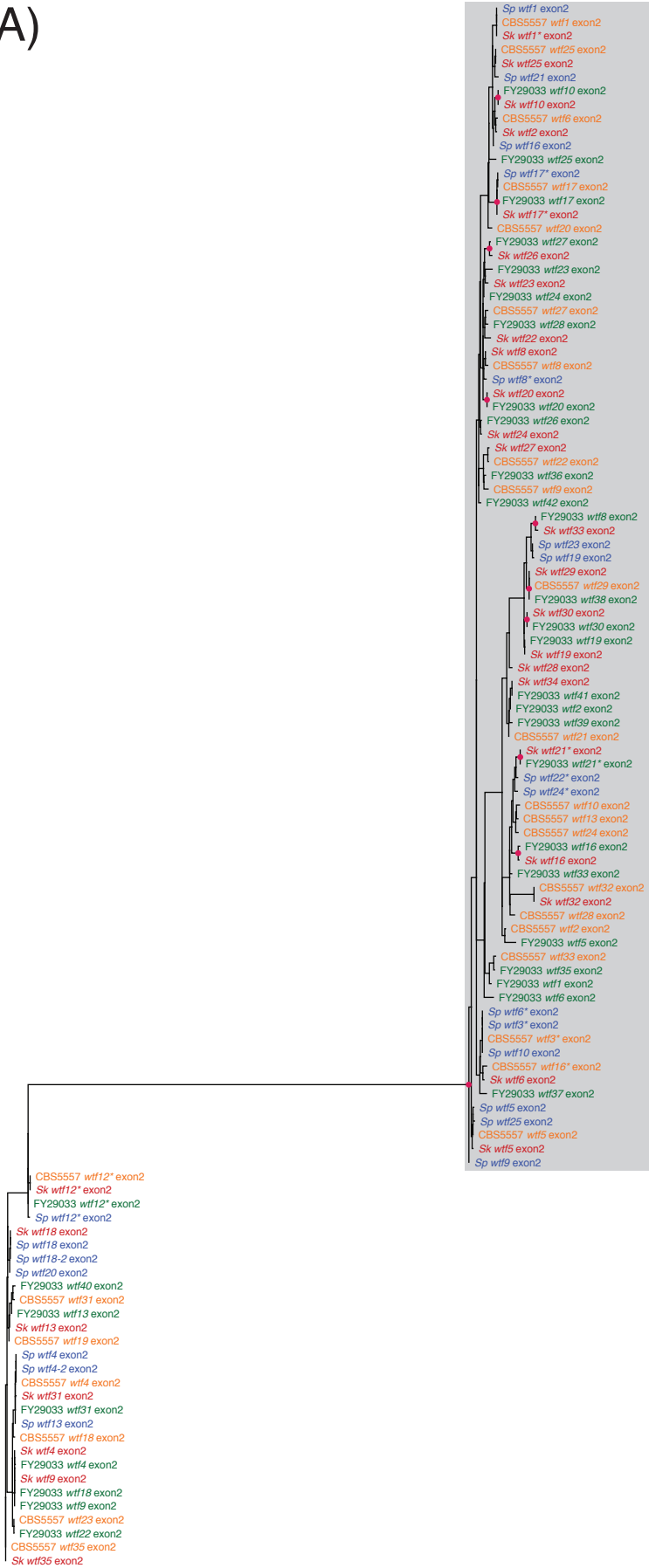

B)

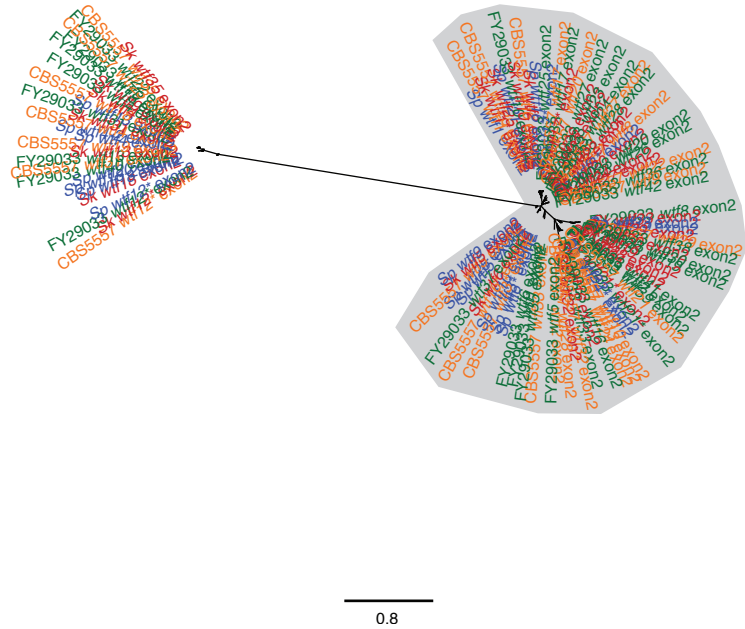

Supplement: Supplementary_Material_msz052 [file supplementary_material_msz052.zip › Supplemental Figure 10 MTEv6.pdf]

A)

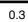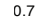

Supplement: Supplementary_Material_msz052 [file supplementary_material_msz052.zip › Supplemental Figure 11 MTEv5.pdf]

A)

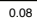

B)

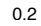

Supplement: Supplementary_Material_msz052 [file supplementary_material_msz052.zip › Supplemental Figure 12 MTEv5.pdf]

A)

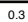

B)

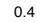

Supplement: Supplementary_Material_msz052 [file supplementary_material_msz052.zip › Supplemental Figure 13 MTEv6.pdf]

# Supplemental Figure 14

A)

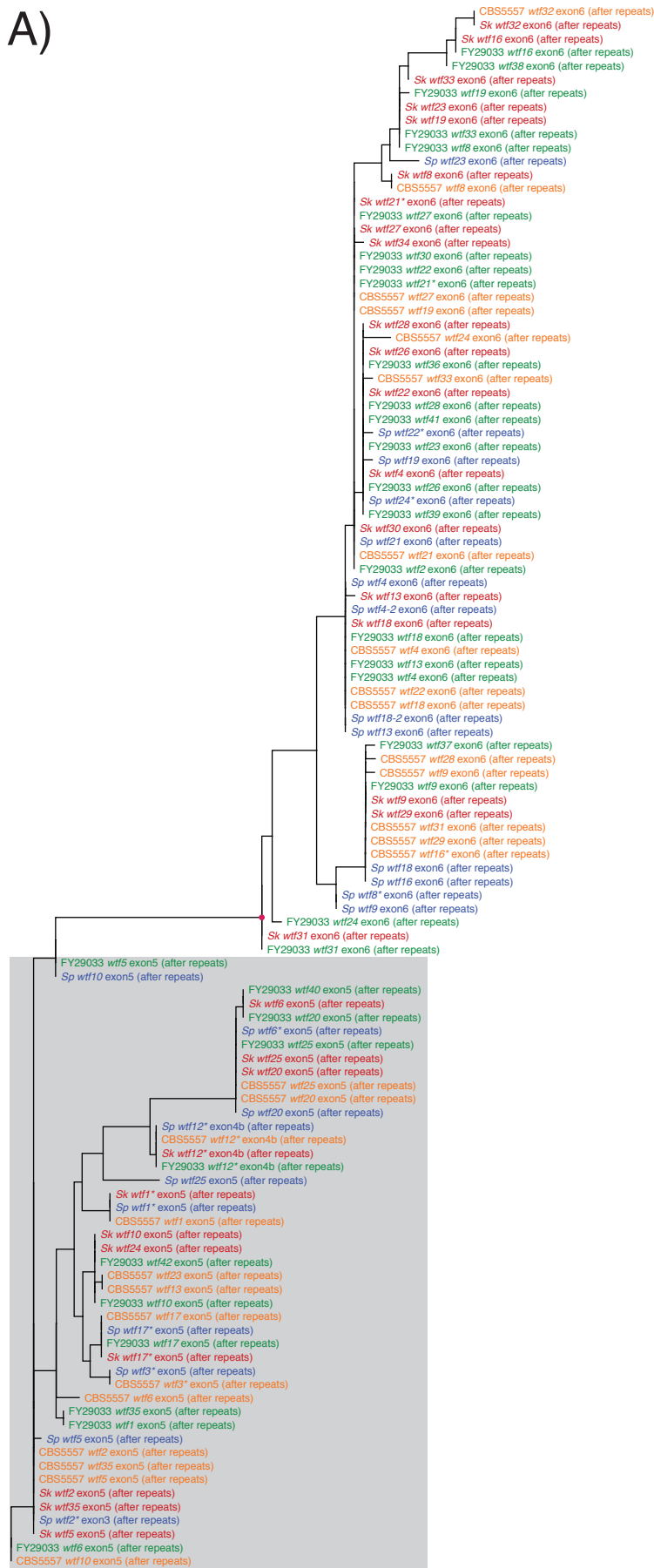

B)

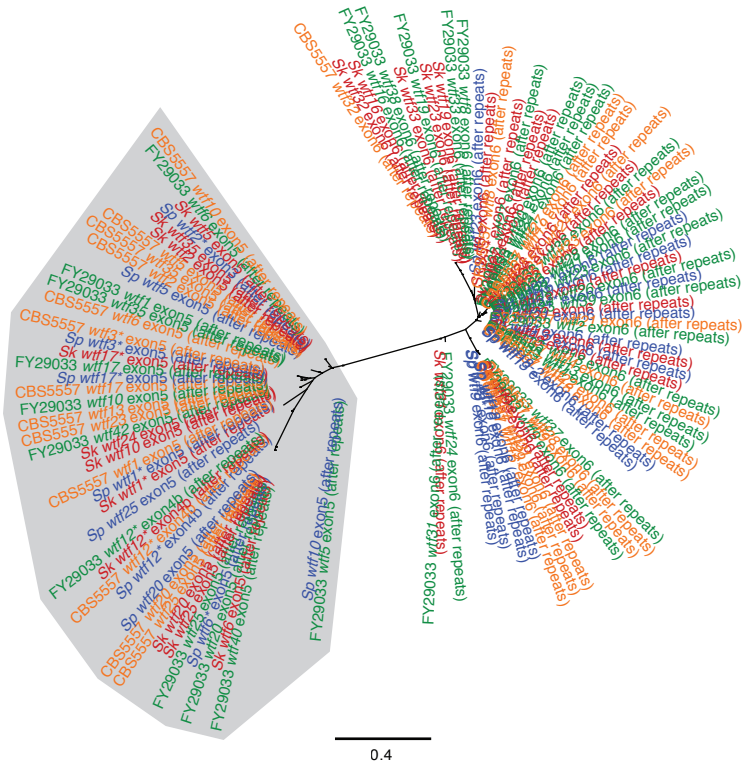

0.4

0.2

Supplement: Supplementary_Material_msz052 [file supplementary_material_msz052.zip › Supplemental Figure 14 MTEv6.pdf]

## Supplemental Figure 15

A)

B)

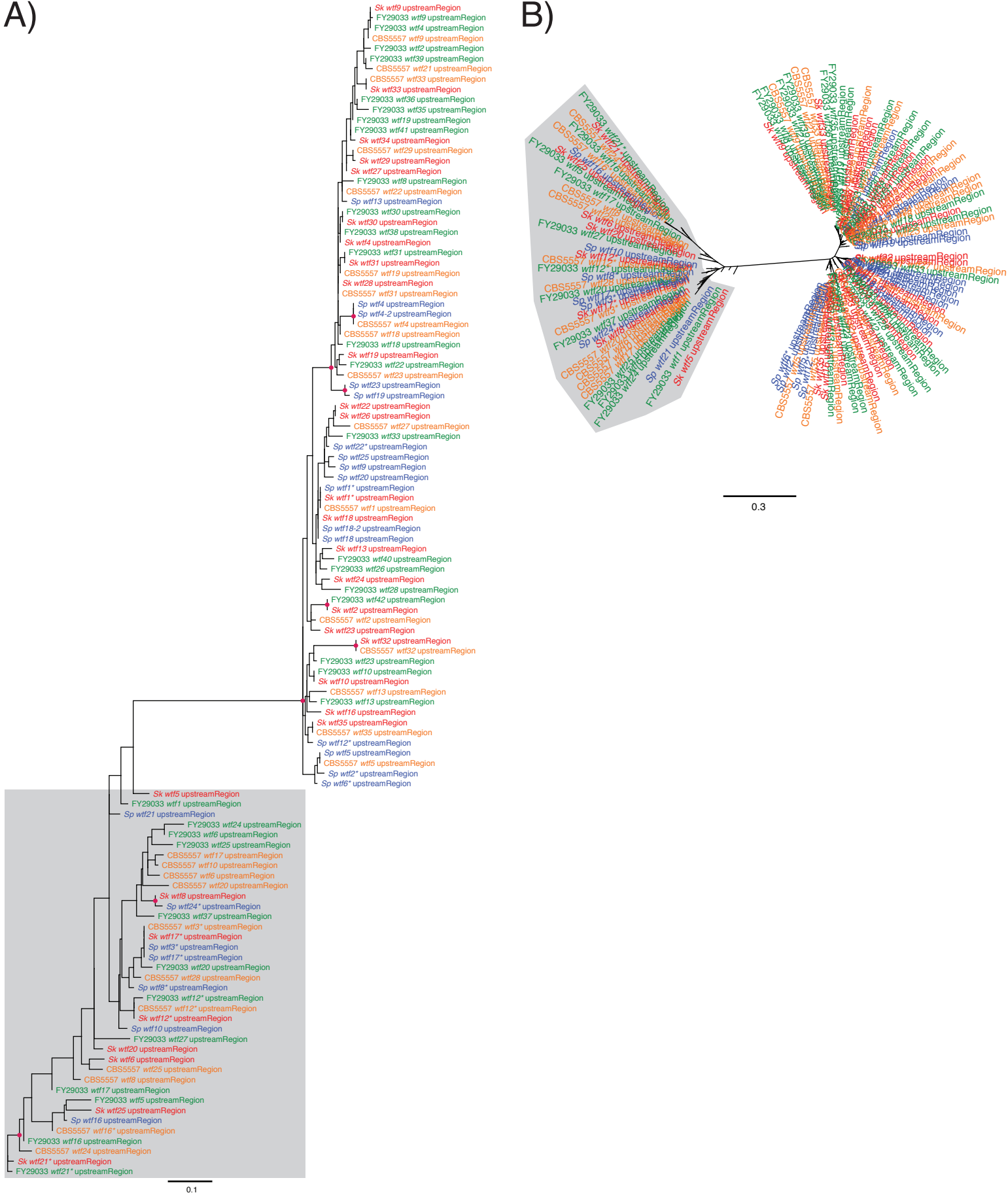

Supplement: Supplementary_Material_msz052 [file supplementary_material_msz052.zip › Supplemental Figure 15 MTEv5.pdf]

Supplemental Figure 16

A)

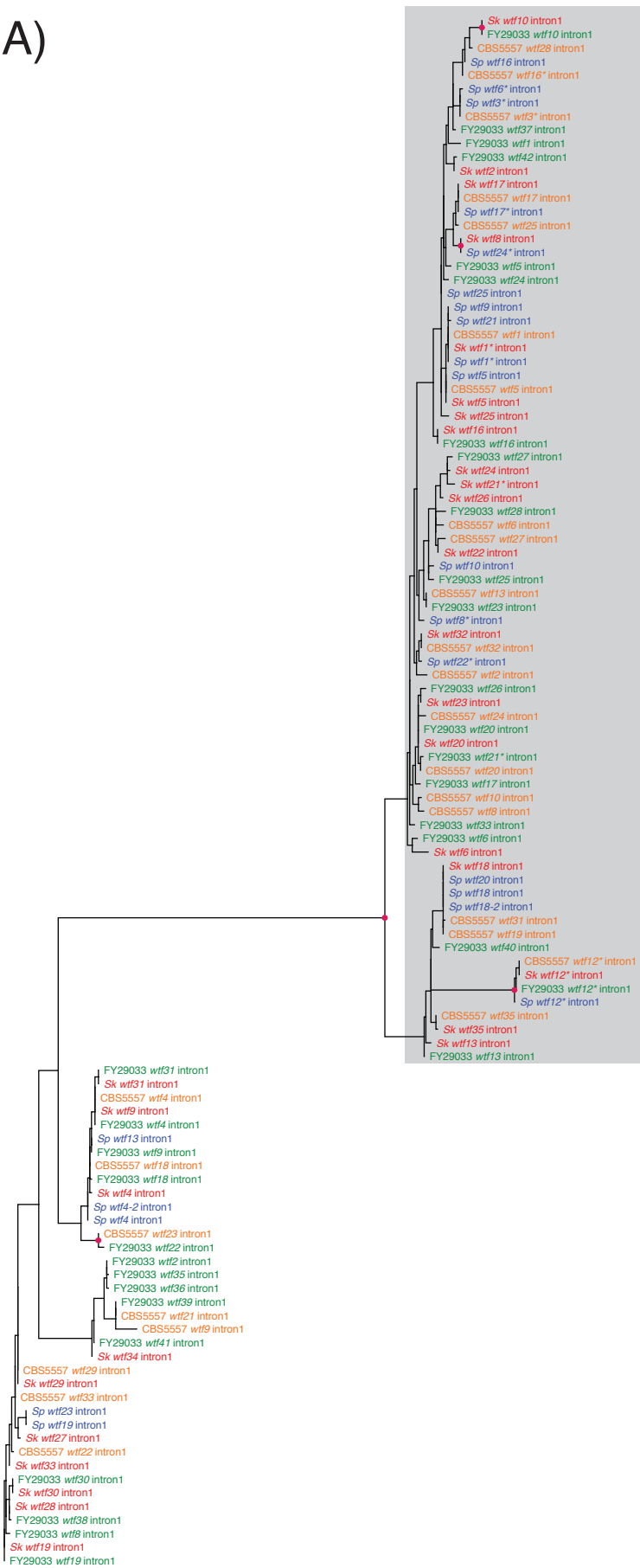

B)

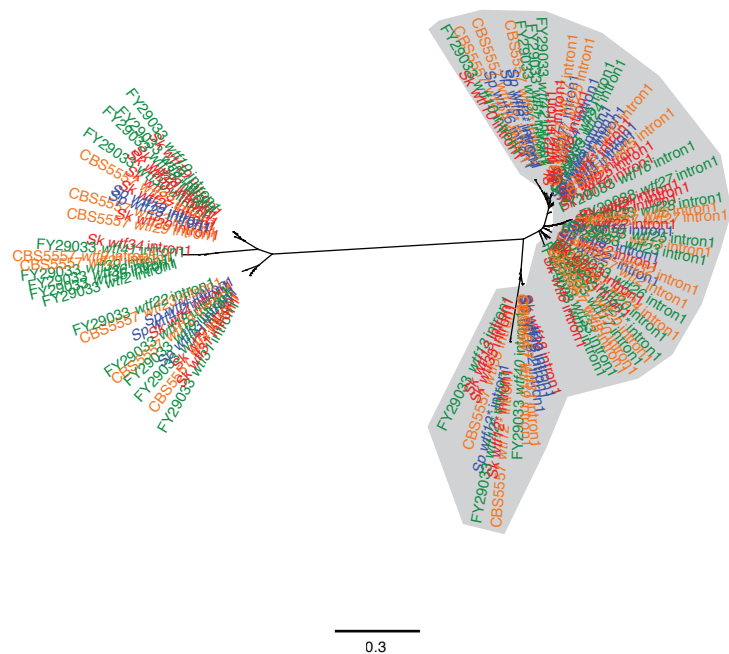

Supplement: Supplementary_Material_msz052 [file supplementary_material_msz052.zip › Supplemental Figure 16 MTEv7.pdf]

# Supplemental Figure 17

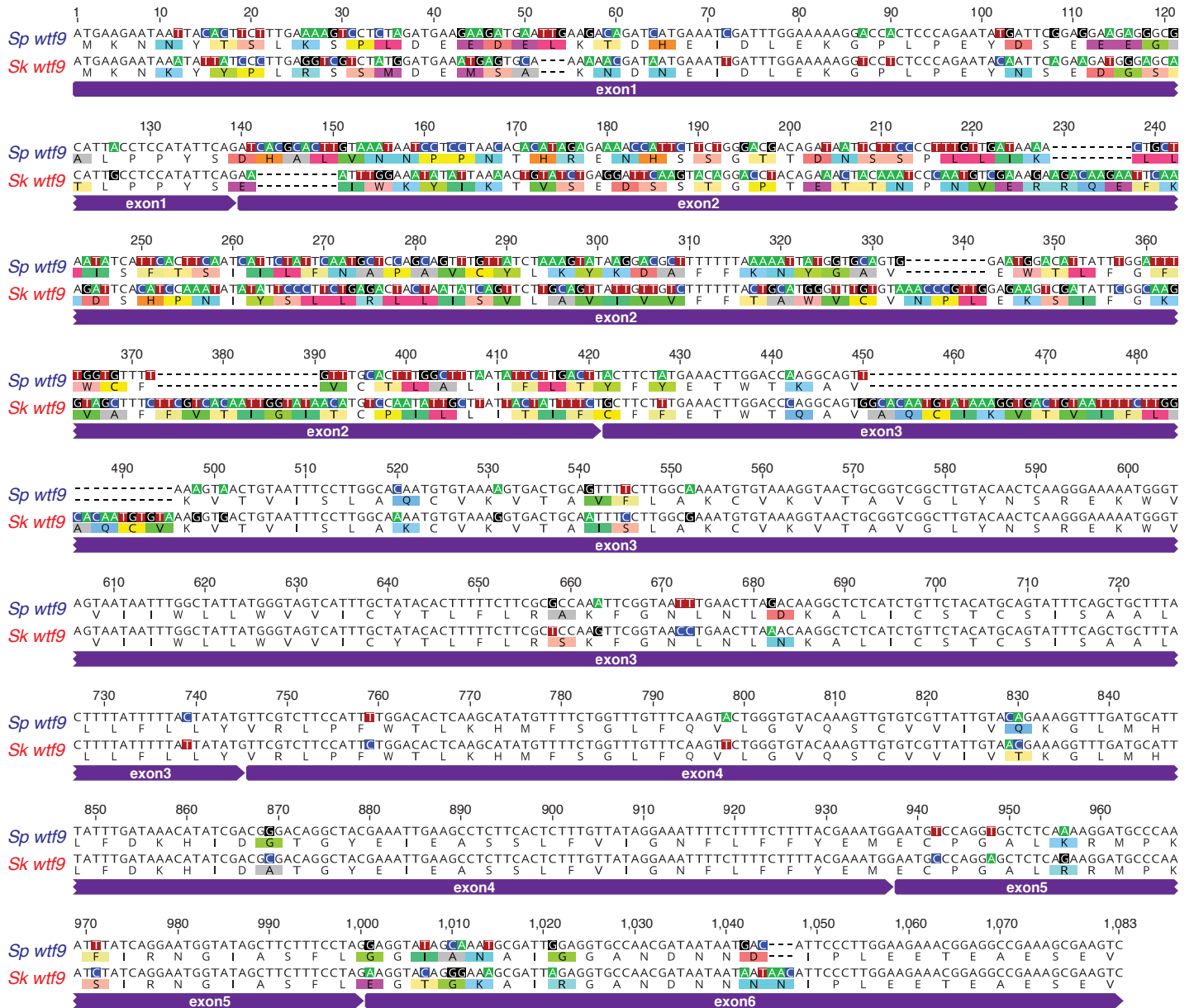

Supplement: Supplementary_Material_msz052 [file supplementary_material_msz052.zip › Supplemental Figure 17 MTEv1.pdf]

Supplemental Figure 2

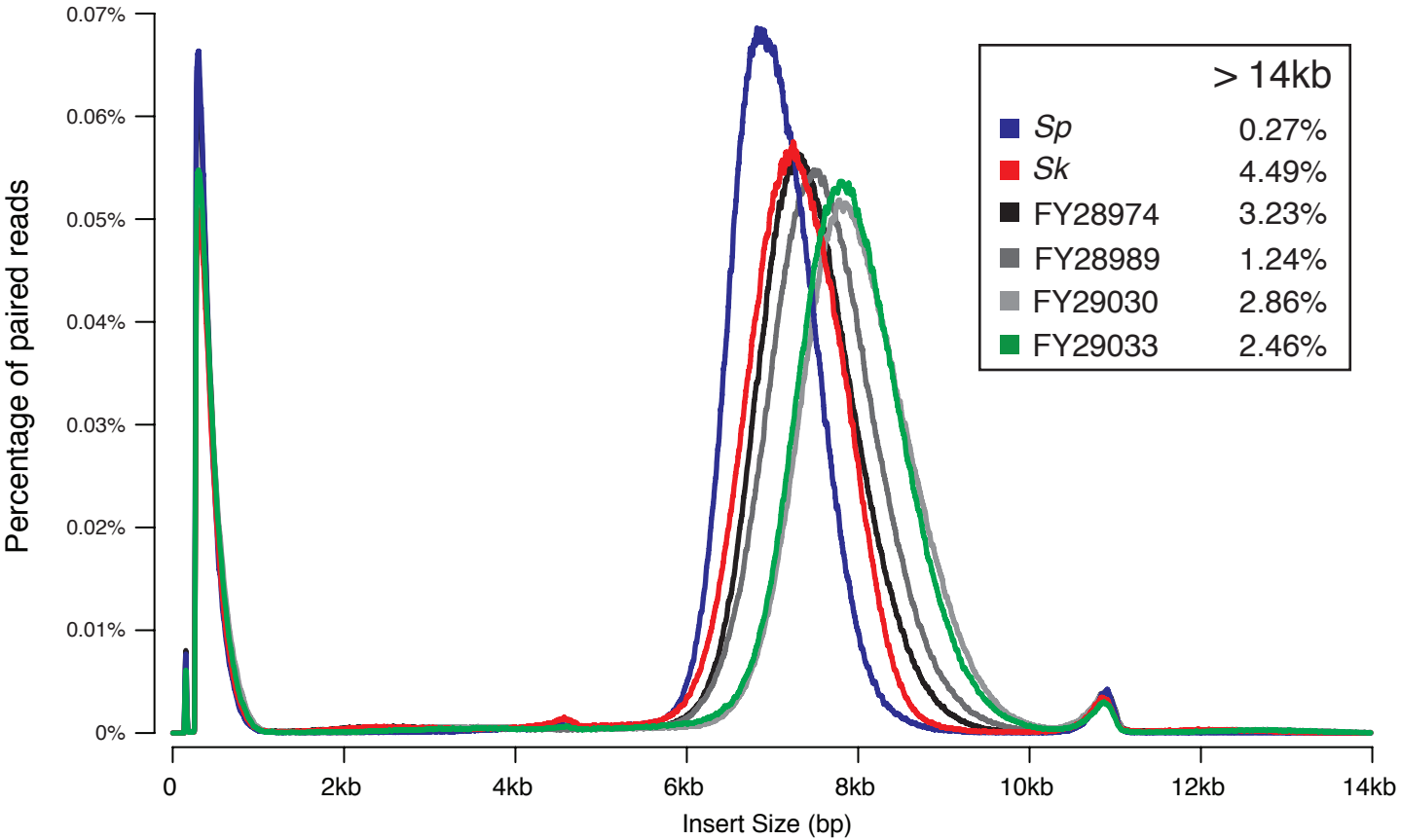

Supplement: Supplementary_Material_msz052 [file supplementary_material_msz052.zip › Supplemental Figure 2 MTEv4.pdf]

Supplemental Figure 3

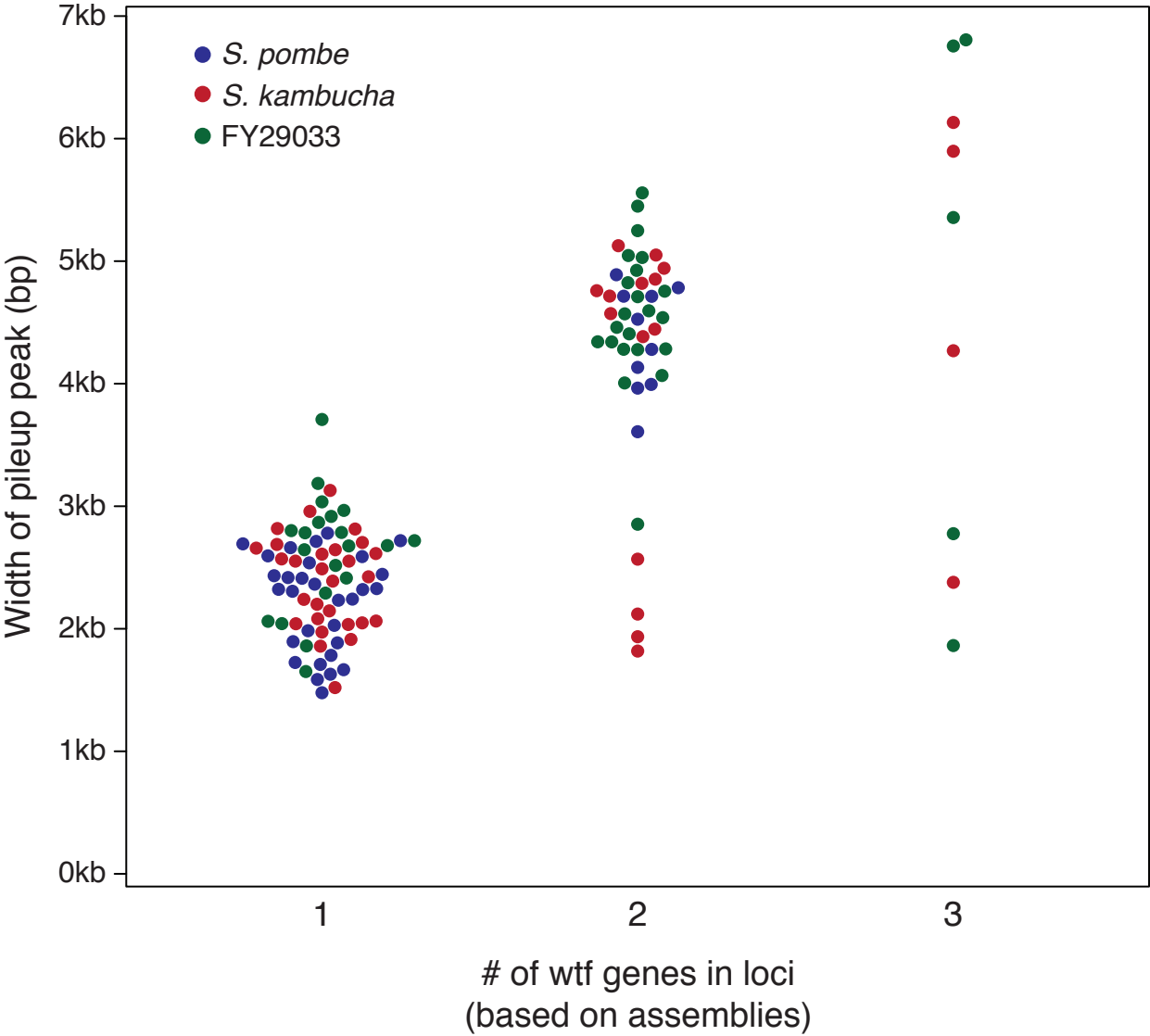

Supplement: Supplementary_Material_msz052 [file supplementary_material_msz052.zip › Supplemental Figure 3 MTEv3.pdf]

## Supplemental Figure 4

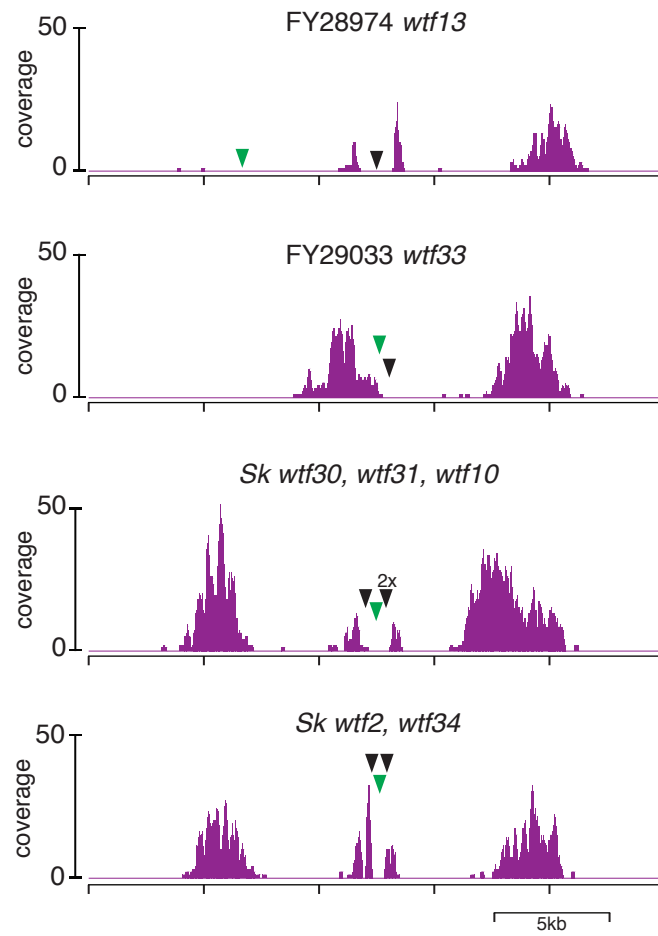

Supplement: Supplementary_Material_msz052 [file supplementary_material_msz052.zip › Supplemental Figure 4 MTEv2.pdf]

Supplemental Figure 5

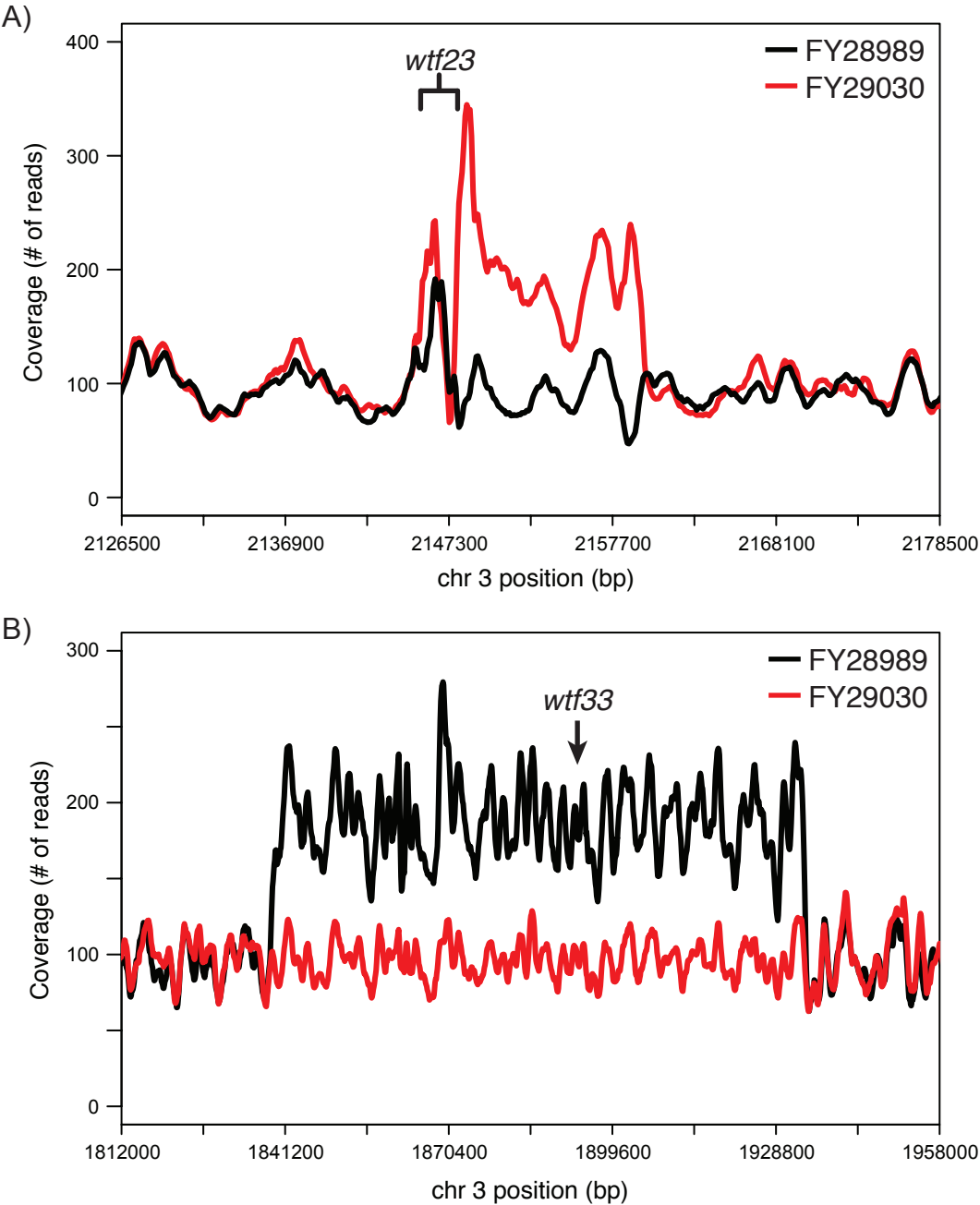

Supplement: Supplementary_Material_msz052 [file supplementary_material_msz052.zip › Supplemental Figure 5 MTEv2.pdf]

# Supplemental Figure 6

***S. pombe***

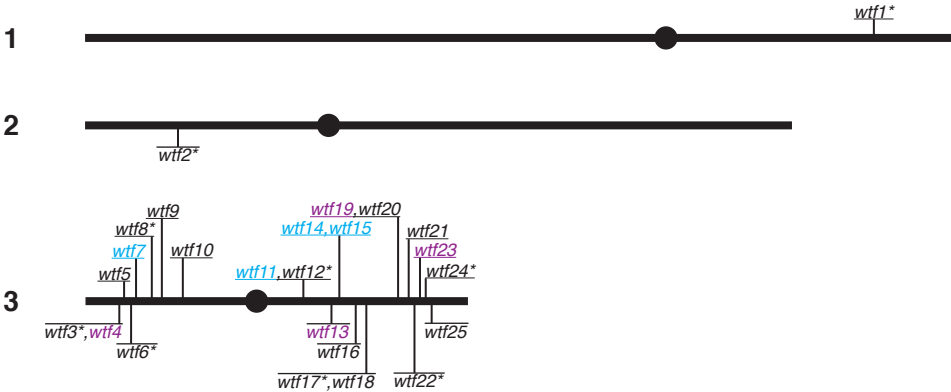

***S. kambucha***

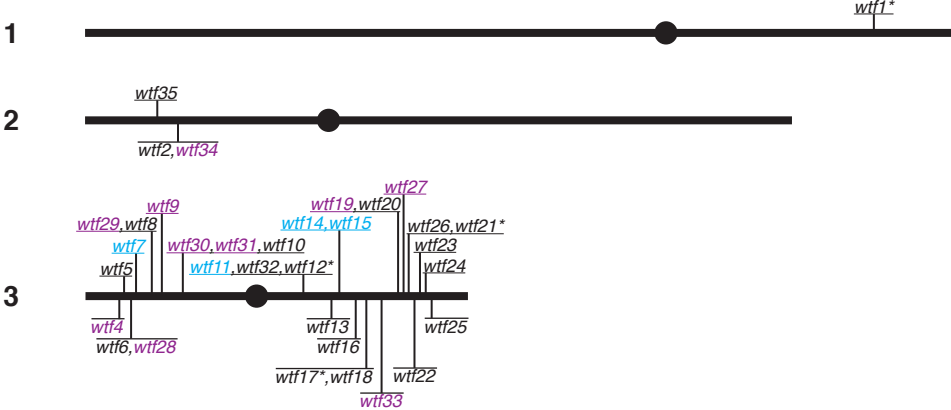

**FY29033**

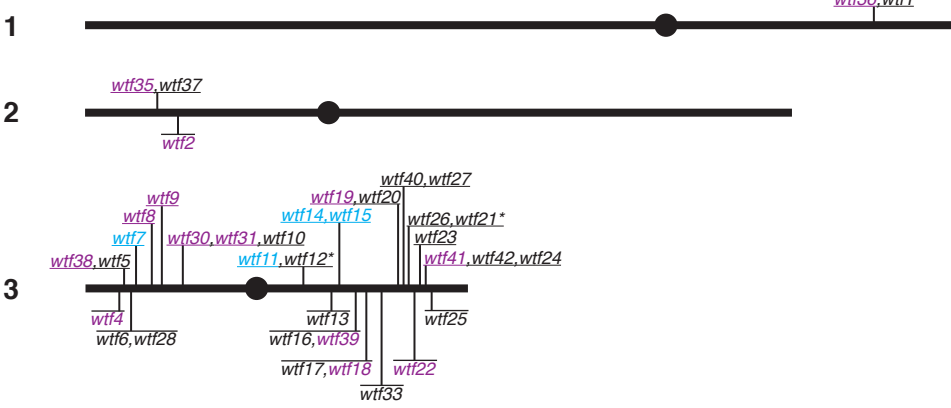

**CBS5557**

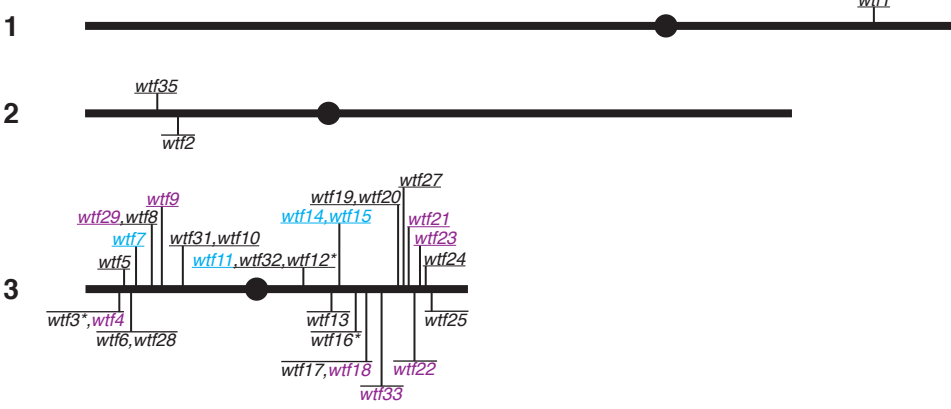

Supplement: Supplementary_Material_msz052 [file supplementary_material_msz052.zip › Supplemental Figure 6 MTEv5.pdf]

Supplemental Figure 7

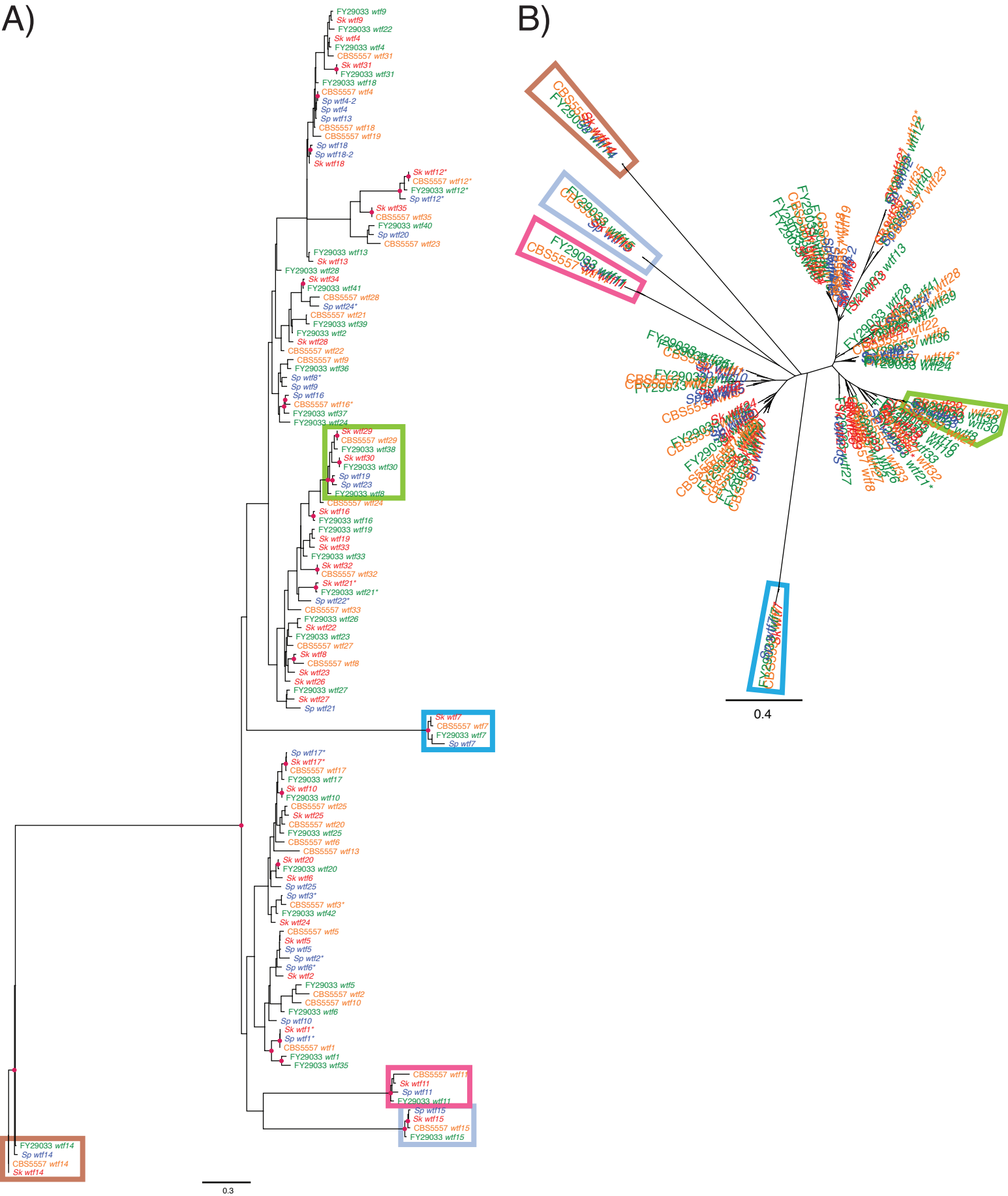

Supplement: Supplementary_Material_msz052 [file supplementary_material_msz052.zip › Supplemental Figure 7 MTEv6.pdf]

# Supplemental Figure 8

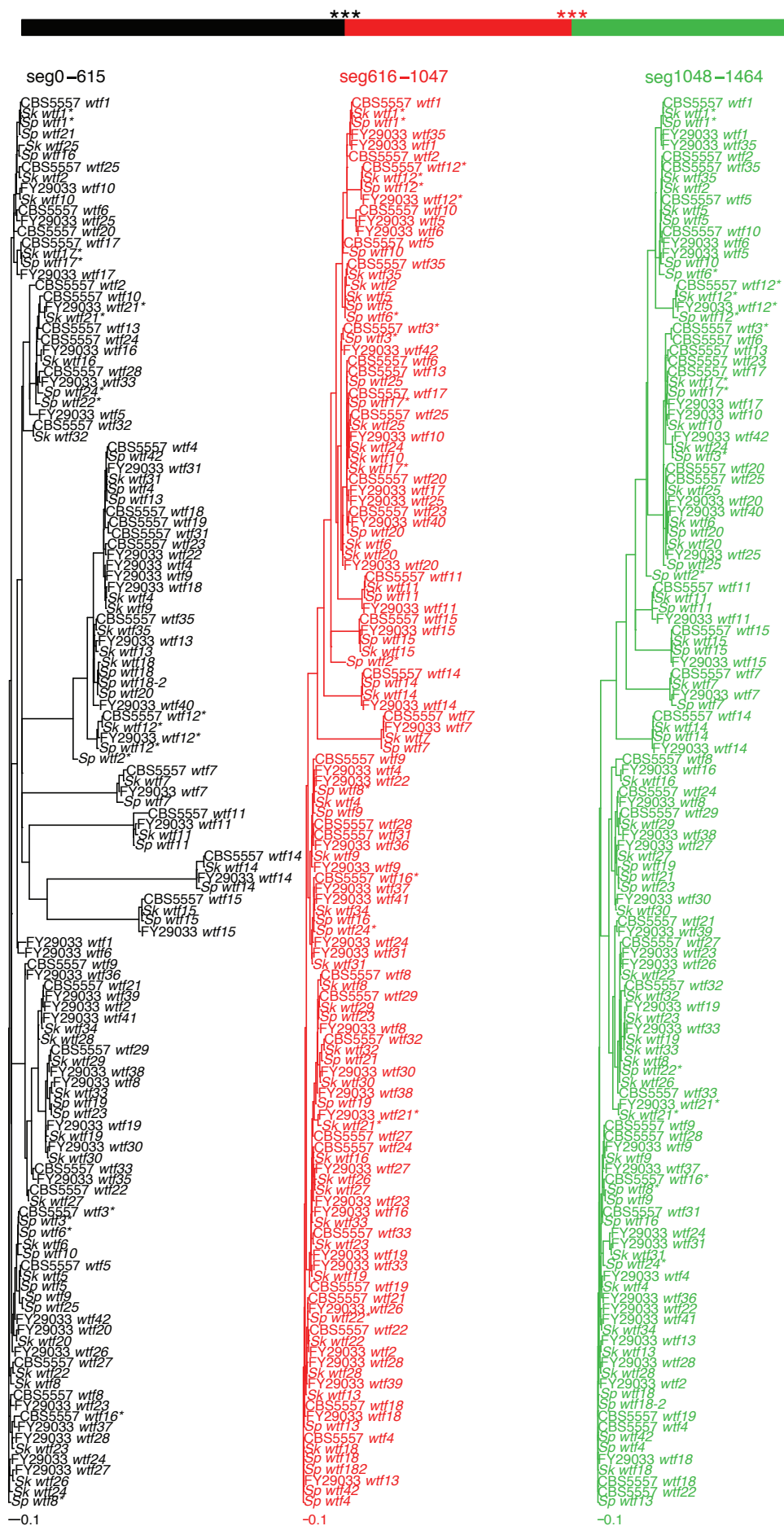

Supplement: Supplementary_Material_msz052 [file supplementary_material_msz052.zip › Supplemental Figure 8 MTEv3.pdf]

Supplemental Figure 9

A)

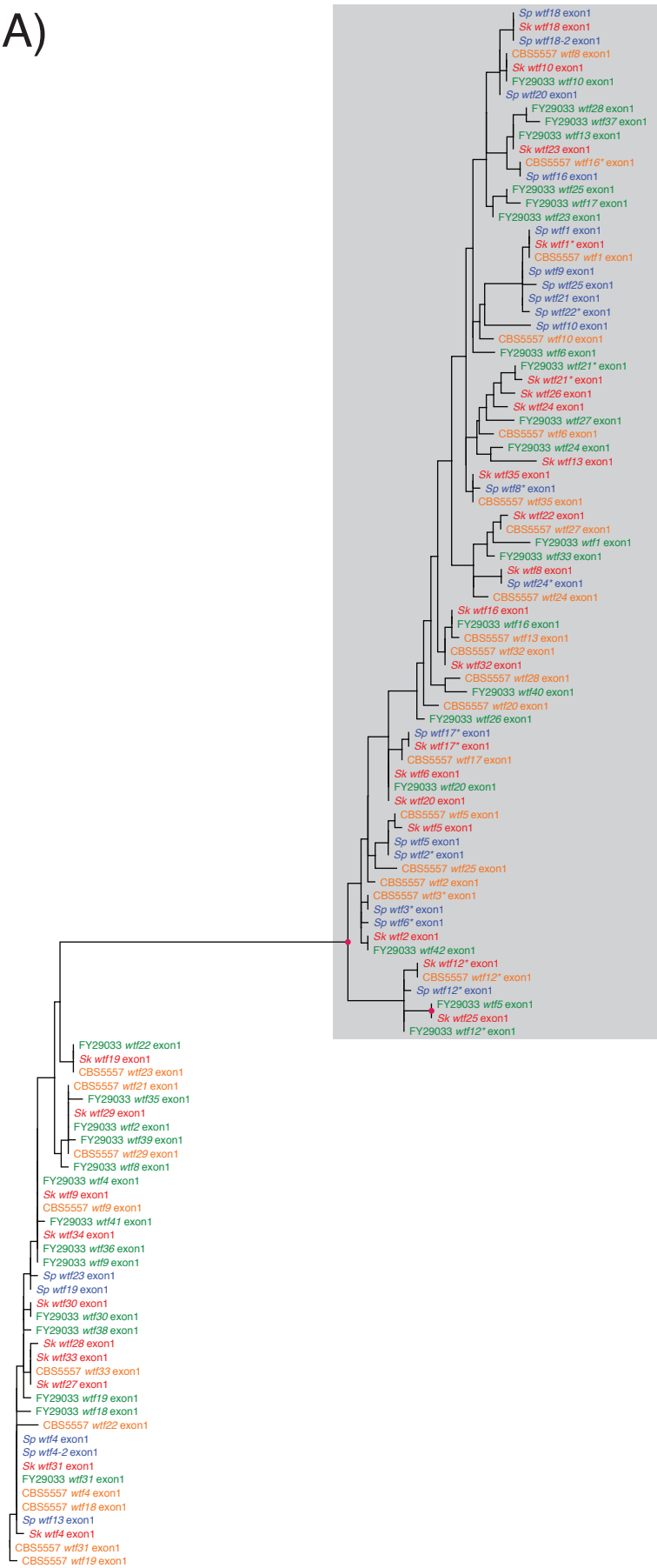

B)

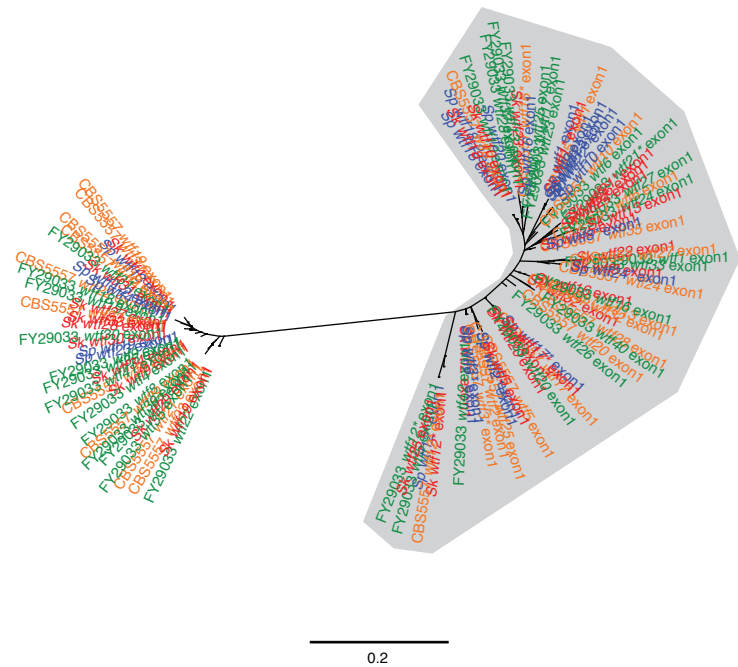

Supplement: Supplementary_Material_msz052 [file supplementary_material_msz052.zip › Supplemental Figure 9 MTEv5.pdf]
